# A Natural Sweetener‐inducible Genetic Switch Controls Therapeutic Protein Expression in Mammals

**DOI:** 10.1002/advs.202514226

**Published:** 2026-01-04

**Authors:** Longliang Qiao, Zhihao Wang, Shasha Tang, Yuan Fang, Guiling Yu, Xiaoting Qiu, Lingxue Niu, Tao Yan, Xingwan Liu, Xiaoding Ma, Deqiang Kong, Yang Zhou, Ningzi Guan, Jinzhong Tian, Meiyan Wang, Haifeng Ye, Fengfeng Cai

**Affiliations:** ^1^ Department of Breast Surgery School of Medicine Tongji Hospital Tongji University Shanghai China; ^2^ Shanghai Frontiers Science Center of Genome Editing and Cell Therapy Biomedical Synthetic Biology Research Center Shanghai Key Laboratory of Regulatory Biology Institute of Biomedical Sciences and School of Life Sciences East China Normal University Shanghai China; ^3^ College of Food and Pharmaceutical Sciences Ningbo University Ningbo China; ^4^ Shanghai Fengxian District Central Hospital Shanghai China; ^5^ Health Science Center Wuhu Hospital East China Normal University Wuhu China; ^6^ Shanghai 411 Hospital，China RongTong Medical Healthcare Group Co.Ltd. ，411 Hospital, School of Medicine Shanghai University Shanghai China

**Keywords:** cell‐based therapies, D‐Psicose cola, gene switch, PURE system

## Abstract

Cell‐based therapies are recognized as the next generation living therapeutics in medicine, especially through the design of synthetic gene switches to enhance the safety and controllability of engineered cells. However, current small molecule‐regulated synthetic gene switches face clinical limitations such as long‐term side effects and metabolic disturbances. Here, we develop a natural sweetener psicose‐inducible transgene expression (PURE) system based on the transcriptional repressor PsiR from *Agrobacterium tumefaciens*. We increase the induction sensitivity of PURE using computational docking to identify candidate PsiR mutations (PsiR_T135N;V134S_), thereby enhancing reporter expression in cell cultures exposed to low psicose concentrations. As a proof‐of‐concept, the designer cells equipped with the PURE system are encapsulated and implanted into the peritoneal cavity of type 1 diabetic (T1D) mice or high‐fat diet (HFD)‐induced obesity model mice. We show that the designer cells could regulate insulin expression to effectively lower blood glucose levels in T1D model mice and induce an anti‐obesity therapeutic protein (thymic stromal lymphopoietin, mTSLP) to reduce body weight in HFD mice, when the psicose‐containing soft drink (psicose cola) is orally administered. This study provides a practical and user‐friendly approach for sustained therapeutic protein delivery in next‐generation cell‐based therapies.

## Graphical abstract

This study develops a natural sweetener, the psicose‐inducible transgene expression (PURE) system based on an *Agrobacterium tumefaciens*–derived transcriptional repressor PsiR. The PURE system is highly specific to psicose, being insensitive to other sugars and structurally similar molecules. As a proof‐of‐concept, we demonstrate that microencapsulated designer cells can be activated by drinking psicose cola to regulate therapeutic protein expression in vivo, achieving glycaemic control in diabetic mice and body weight reduction in obese mice.

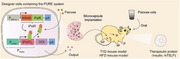

## 1 Background

Cell‐based therapies broadly follow the sense and respond pattern, in which they rely on designer cells that sense a user‐defined input signal, process it, and respond appropriately through a customizable therapeutic output [1, 2, 3]. Precise regulation over the dosage and timing of the therapeutic program is critical to enhance safety and efficacy outcomes with minimal side effects and to facilitate successful clinical translation of next‐generation gene‐ or cell‐based therapies [4, 5]. To date, a multitude of trigger‐inducible transgene expression systems have been developed, which are responsive to various inducer molecules for use in mammalian cells and transgenic animals [6, 7], such as antibiotics [8, 9], vitamins [10], plant‐derived compounds [11, 12, 13, 14], food additives [15], or cosmetics [16]. However, these trigger molecules have their own problems, including antibiotic resistance, unintentional activation by exposures occurring during everyday life, lack of safety and orthogonality, or poor patient compliance [17, 18, 19].

The expected candidate trigger molecules for clinical and/or biomedical applications would be natural, nontoxic, highly soluble, and inexpensive [20]. D‐Psicose (psicose) is a low‐calorie epimer of the monosaccharide sugar fructose, used by some major commercial food and beverage manufacturers as a low‐calorie sweetener, and its sweetness is estimated to be 70% of sucrose [21]. The U.S. Food and Drug Administration (FDA) has accepted a petition for Generally Recognized as Safe (GRAS) status for psicose as a sugar substitute in various specified food categories (GRN No. 400). Psicose is metabolized at an extremely low rate in vivo, indicating that it is not readily broken down by cellular enzymes to generate energy like conventional sugars [22], and it is minimally absorbed and does not raise insulin levels [23]. Thus, psicose represents an attractive inducer for a trigger‐inducible transgene expression system.

Here, we designed a beverage‐triggered, remote‐control switch triggered by psicose and tested it in mammals. First, the psicose‐inducible transgene expression (PURE) was designed on the basis of a transcriptional repressor PsiR [24], derived from *Agrobacterium tumefaciens* and fused to a trans‐silencing Krueppel‐associated box (KRAB) domain, which specifically binds to synthetic cognate promoters containing PsiR‐binding sites. PsiR is a LacI‐family transcriptional regulator with high affinity for psicose, which enables binding a consensus sequence in the promoter region and prevents transcription of the regulated promoters in the absence of psicose [24]. Next, we analyzed the structure of the PsiR and psicose binding states using RosettaFold and AutoDock, and developed a sensitive and more efficiently inducible PURE system based on rational mutation of amino acids in the binding pocket region. We demonstrated that the PURE system is readily and dose‐dependently activated by psicose in mammalian cells and mice. Moreover, psicose was delivered via a sugar‐free cola beverage to activate the PURE system in vivo. In a type 1 diabetes (T1D) mouse model, this approach enabled controlled insulin expression and effectively lowered blood glucose levels. In a high‐fat diet (HFD)‐induced obesity model, psicose‐induced expression of the therapeutic cytokine thymic stromal lymphopoietin (mTSLP) resulted in marked reductions in body weight. By leveraging a widely consumed inducer, this beverage‐derived psicose‐responsive system provides a robust and clinically compatible platform for programming therapeutic gene expression. Such an approach holds promise for enhancing the safety, tunability, and translational potential of next‐generation gene‐ and cell‐based precision medicine.

## 2 Results

### 2.1 Design and Optimization of the PURE System for Transgene Expression Induction

**FIGURE 1:**
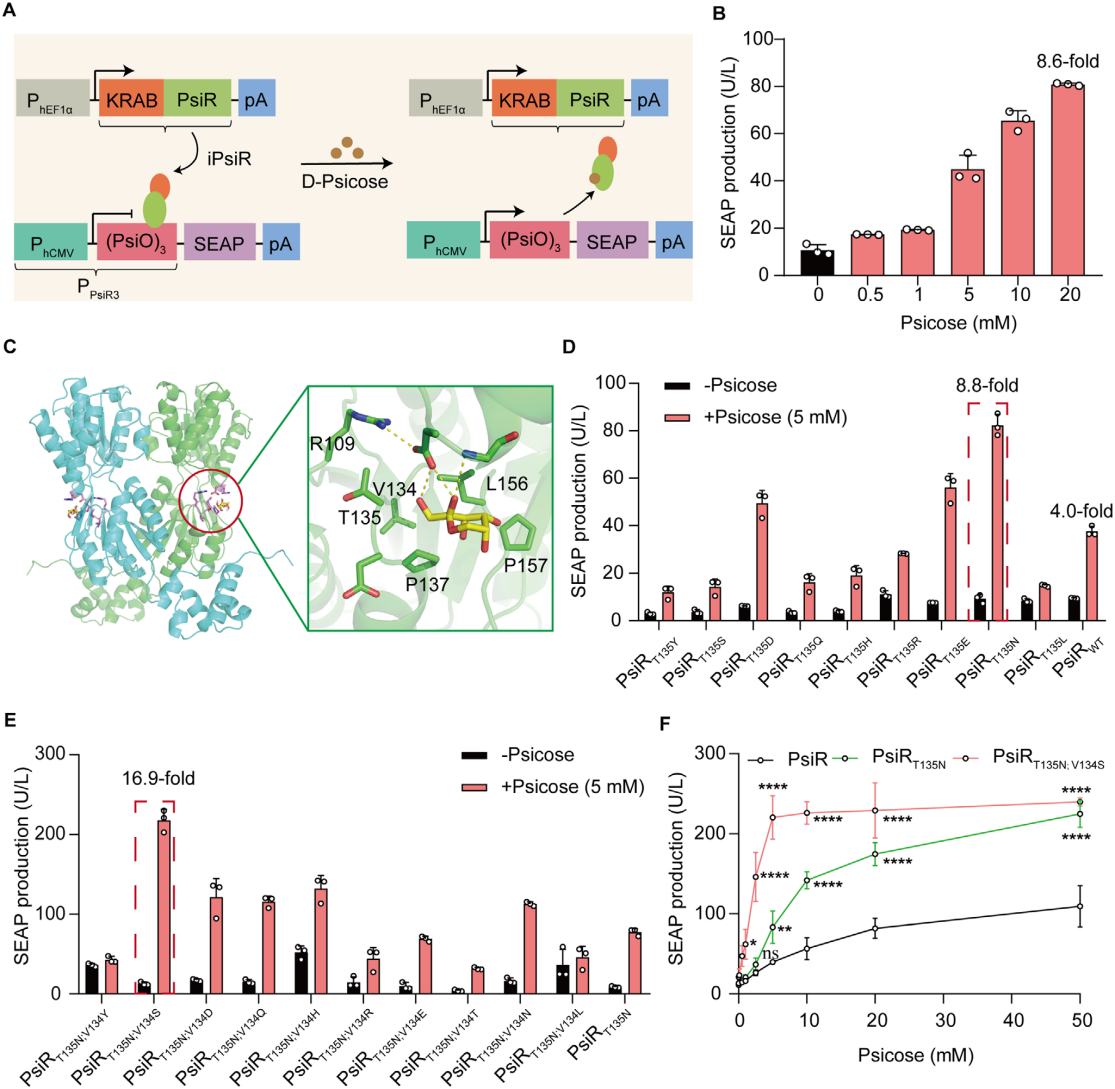
Design and optimization of the PURE system for controlling gene expression in mammalian cells. (A) Schematic showing the design of the PURE system for controlling gene expression. The synthetic mammalian psicose‐triggered transrepressor iPsiR (KRAB‐PsiR) is an N‐terminal of PsiR fused with a trans‐silencing Krüeppel‐associated box (KRAB) domain, which is constitutively expressed by the human elongation factor 1 alpha promoter (P_hEF1α_). In the absence of psicose, iPsiR binds to a chimeric target promoter P_PsiR3_ [P_hCMV_‐(PsiO)_3_] and represses reporter gene SEAP expression; in the presence of psicose, iPsiR is released from P_PsiR3_ and initiates reporter gene SEAP expression. (B) Dose‐dependent psicose‐induced SEAP expression by the PURE system. HEK‐293T cells were co‐transfected with pQL172 (P_hEF1α_‐KRAB‐PsiR‐pA) and pQL164 (P_PsiR3_‐SEAP‐pA). SEAP production was quantified 48 h after cultivation with different concentrations of psicose. (C) The global structure (left) and ligand binding pocket (right) of PsiR in complex with psicose predicted by RosettaFold and AutoDock 4.2. The labeled residues were targeted for mutagenesis except for R109. (D) Performance of the PURE system was evaluated by replacing the 135th amino acid (T135) of wild‐type PsiR (PsiR_WT_) with branched‐chain or polar amino acids: Y, S, D, Q, H, R, E, N, and L. HEK‐293T cells were co‐transfected with pQL164, nine different KRAB‐PsiR_T135_ mutants or PsiR_WT_. SEAP production was quantified 48 h after cultivation with 5 mm psicose. (E) Performance of the PURE system was evaluated by replacing the 134th amino acid (V134) of the PsiR_T135N_ mutant with branched‐chain or polar amino acids: Y, S, D, Q, H, R, E, T, N, L, and T. HEK‐293T cells were co‐transfected with pQL164, different KRAB‐PsiR_T135N;V134_ mutants, or PsiR_T135N_. SEAP production was quantified 48 h after cultivation with 5 mm psicose. (F) Dose‐dependent psicose‐induced SEAP expression by PsiR_WT_, PsiR_T135N_, PsiR_T135N;V134S_. HEK‐293T cells were co‐transfected with pQL164 and either pQL172, or pQL173 (P_hEF1α_‐KRAB‐PsiR_T135N;V134S_‐pA), or pQL174 (P_hEF1α_‐KRAB‐PsiR_T135N_‐pA). SEAP production was quantified 48 h after cultivation with different concentrations of psicose. Data in (B,D–F) are presented as means ± SD; *n* = 3 independent experiments. *P* values in (F) were calculated by one‐way ANOVA with multiple comparisons. ns, not significant, ^*^p < 0.05, ^**^p < 0.01, ^****^p < 0.0001. Detailed descriptions of the genetic constructs and transfection mixtures are provided in Tables S1 and S12.

Capitalizing on psicose‐responsive PsiR, a LacI family transcription factor with high affinity for psicose [24], we engineered a psicose‐inducible transgene (PURE) system in mammalian cells. The PURE system consists of a synthetic mammalian transrepressor, iPsiR (KRAB‐PsiR), generated by fusing the KRAB domain—a well‐characterized transcriptional repression module that recruits co‐repressors and epigenetic modifiers to silence target gene expression [25] with the N terminus of the transcriptional repressor PsiR derived from *Agrobacterium tumefaciens* (Figure 1A). iPsiR binds and represses constitutive gene expression from the synthetic promoter P_PsiR3_ that consists of a tandem iPsiR binding site (operator sequence of PsiO) positioned behind the human cytomegalovirus promoter (P_hCMV_). The PsiO sequence originates from the native promoter pPsiA and is specifically recognized by PsiR. When psicose is present, it disrupts the iPsiR‐dependent repression of PisO, thereby triggering transgene expression (Figure 1A).

To obtain an optimal PURE system permitting minimal basal transgene expression in the absence of psicose and maximal induction rates in the presence of psicose, we first constructed the synthetic PsiR‐specific promoters with a single PsiO copy and found there was a high basal leakage without psicose administration (Figure S1A). Previous studies demonstrated that increasing the number of operator copies can be developed to reduce the background activity by improving repressor binding between the promoter and the reporter gene [11]. Hence, we further designed four different synthetic PsiR‐specific promoters with various iterations of PisO tandem repeats. Among these designs, P_PsiR3_ showed the best fold induction of secreted embryonic alkaline phosphatase (SEAP) expression upon psicose stimulation, achieving an optimal balance between minimal basal activity and maximal inducibility (Figure 1B; Figure S1). However, it had a strong background without 20 mm psicose and low SEAP reporter gene activation (8.6‐fold) with 20 mm psicose (Figure 1B).

Seeking to decrease the amount of psicose needed to trigger the PURE system facilitating biomedical applications, we were motivated to improve the sensitivity of the PURE system. First, the overall structure of PsiR was predicted using the online software RosettaFold (robetta.bakerlab.org), and then PsiR was docked with psicose using AutoDock 4.2 [26] (Figure 1C). Molecular docking showed that five amino acids (T135, V134, P137, L156, P157) in close proximity to psicose are potential candidates for PsiR protein modification (Figure 1C). In addition, a previous study had demonstrated that an amino acid with a branched chain substitutions introduced new hydrogen‐bonding interactions between transcriptional repressors and a chemical molecule [27], which improved the response sensitivity of the transcriptional repressors. First, we generated PsiR mutants where the 135th amino acid (T135) of PsiR was replaced with branched‐chain or polar amino acids: Y, S, D, Q, H, R, E, N, and L, to assess their effects on protein function. PsiR_T135N_ showed the highest induction efficiency (8.8‐fold) of SEAP expression, while a 4.4‐fold induction was achieved with wild‐type PsiR in the presence of 5 mm psicose (Figure 1D). The results indicated that the PsiR_T135N_ mutant exhibited significantly higher response sensitivity to psicose.

In the second round, we generated PsiR_T135N_ mutants where the134th residue (V134) of the PsiR_T135N_ mutant was replaced with Y, S, D, Q, H, R, E, T, N, L, and T. PsiR_T135N;V134S_ variant had a highest induction efficiency (16.9‐fold) of SEAP reporter expression in the presence of 5 mm psicose (Figure 1E). After this, the 137th, 156th, and 157th amino acid (P137, L156, P157) of the PsiR_T135N;V134S_ variant were further replaced with the above branched‐chain or polar amino acids. However, the third‐round mutations did not further improve the fold‐induction of SEAP reporter expression in the presence of 5 mm psicose (Figure S2A–C).

Then, three different versions of iPsiR (PsiR_WT_, PsiR_T135N_, PsiR_T135N;V134S_) were selected to be transfected with HEK‐293T cells and exposed to psicose (from 0 to 50 mm). The findings showed that a mutated PsiR_T135N;V134S_ significantly increased reporter gene expression in cell cultures treated with low psicose concentration (5 mm psicose) compared to a mutated PsiR_T135N_ and WT PsiR, and the SEAP signal reached a saturation level in three different versions of PsiR after treatment of 20 mm psicose (Figure 1F).

In addition, we have evaluated the dose‐dependent psicose‐induced SEAP expression using wild‐type PsiR, the previously reported PsiR_G133D;Y233R_ mutant [27], and our PsiR_T135N;V134S_ variant. The results showed that PsiR_G133D;Y233R_ mutant had no reporter gene expression in the presence of psicose (0–50 mm), while PsiR_T135N;V134S_ variant had the highest induction efficiency of SEAP expression. This suggested that the PsiR_G133D;Y233R_ mutant failed to respond to psicose under our experimental conditions (Figure S3) although it efficiently bound to the PsiO operator and repressed reporter expression. We speculated that this discrepancy may arise from different assay design, host background, or experimental context.

### 2.2 Characterization of the PURE System

**FIGURE 2:**
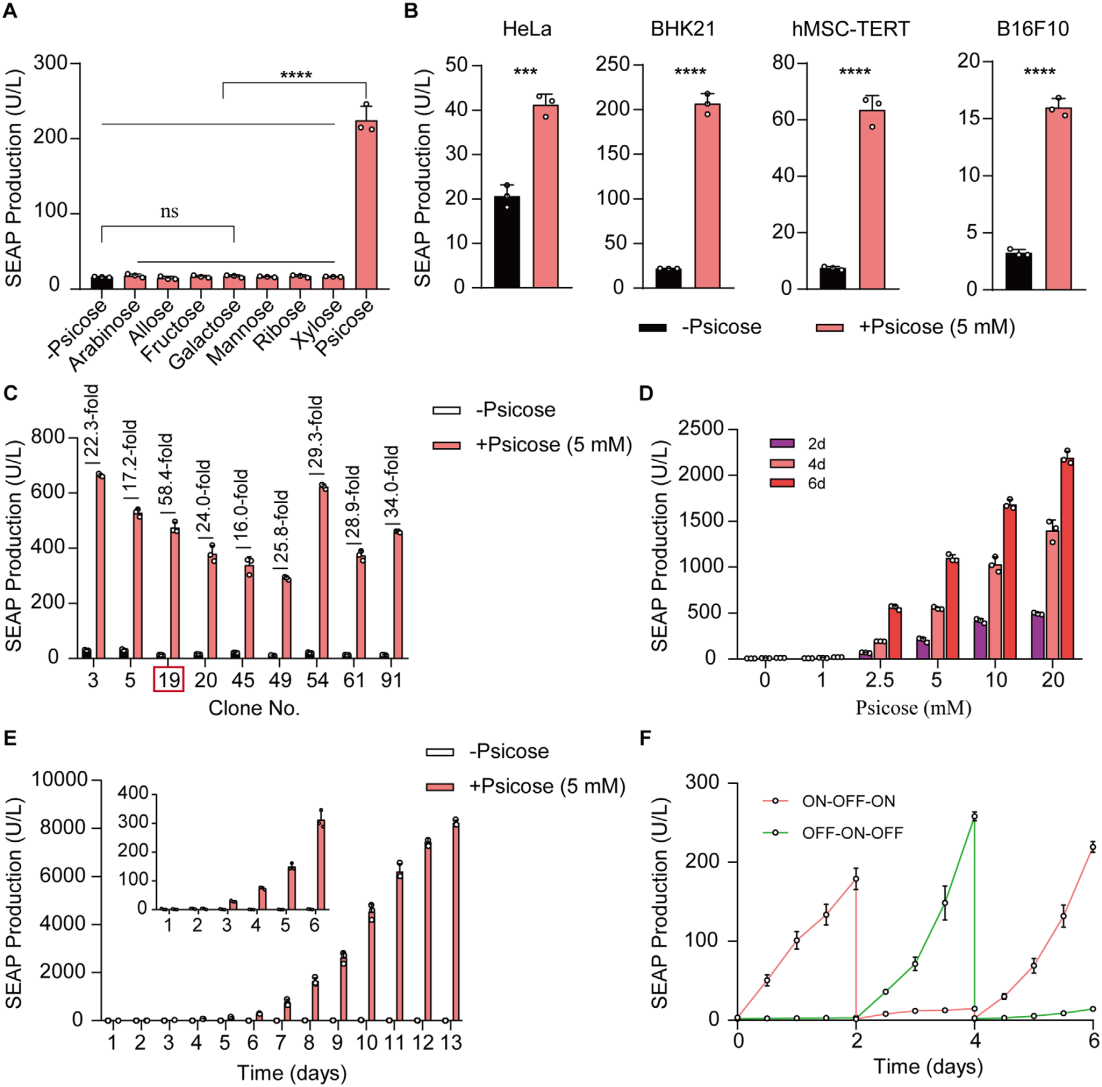
Characterization of the PURE system in mammalian cells. (A) Specificity of the PURE system for psicose. HEK‐293T cells co‐transfected with pQL164 and pQL173 (P_hEF1α_‐KRAB‐PsiR_T135N;V134S_‐pA), SEAP production was quantified 48 h after cultivation with 5 mm of one of the following sugars: arabinose, allose, fructose, galactose, mannose, ribose, xylose, and psicose. (B) PURE‐mediated SEAP expression in the indicated mammalian cell lines co‐transfected as described in (B) after cultivating for 48 h in the presence or absence of psicose. (C) Selection of stably transgenic psicose‐inducible cell lines (HEK_PURE‐SEAP_). HEK293T cells were co‐transfected with pQL451 (ITR‐P_PsiR3_‐SEAP‐P2A‐mINS‐pA::P_mPGK_‐ZeoR‐pA‐ITR), pQL450 (ITR‐P_hEF1α_‐KRAB‐PsiR_T135N;V134S_‐pA::P_mPGK_‐PuroR‐pA‐ITR), and the transposase expression vector pCMV‐T7‐SB100 (P_hCMV_‐SB100X‐pA). The selected cell clones were profiled for their psicose‐inducible SEAP expression. The red frame marks the best‐in‐class clone (Clone No.19, 58.4‐fold) chosen for the following experiments. (D) Dose‐dependent SEAP expression in HEK_PURE‐SEAP_ stable cells after cultivating in complete medium supplemented with different concentrations of psicose (0–20 mm) for 2, 4 and 6 days. (E) Long‐term SEAP production kinetics in HEK_PURE‐SEAP_ stable cells. HEK_PURE‐SEAP_ cells (1 × 10^5^) were seeded in a 10 cm‐dish and incubated with or without 5 mm psicose for 13 days. The culture medium was replaced with fresh medium every 48 h, and SEAP production was profiled every 24 h. (F) Reversibility of HEK_PURE‐SEAP_‐mediated SEAP expression. HEK_PURE‐SEAP_ cells (5 × 10^4^) were cultivated for 6 days while alternating the psicose concentrations from 0 to 2.5 mm, and SEAP expression in the culture supernatants was profiled every 12 h. Cell density was adjusted to 5 × 10^4^ every 2 days. Data are presented as means ± SD; *n* = 3 independent experiments. *P* values in A were calculated by one‐way ANOVA with multiple comparisons. *P* values in (B, C) were calculated using a two‐tailed unpaired *t*‐test. ns, not significant, ^***^p < 0.001, ^****^p < 0.0001. Detailed descriptions of the genetic constructs and transfection mixtures are provided in Tables S1 and S2.

We next evaluated the functional performance of the PURE system. First, we tested whether a set of different sugars and structurally similar molecules could activate SEAP reporter gene expression, including psicose, allose, fructose, galactose, mannose, ribose, xylose, and arabinose. We found that an obvious SEAP signal was only observed in cells after exposure to psicose; no signal was evident for cells after exposure to other screened compounds (Figure 2A), suggesting the PURE system is highly specific for psicose as an inducer.

To demonstrate the use of our system beyond HEK‐293T cells, we transiently transfected the PURE system and demonstrated that our system worked in a range of rodent and mammalian cell lines including human cervical adenocarcinoma cells (HeLa), baby hamster kidney cells (BHK‐21), human telomerase‐immortalized mesenchymal stem cells (hMSC‐TERT), and mouse melanoma cells (B16F10) (Figure 2B). However, different cells transfected with the PURE system showed activation of SEAP expression with different performance in the presence of psicose. The different response characteristics among different cells could probably be cell specific factors, including (i) differences in transfection efficiency, (ii) variations in chromatin structure that affect synthetic promoter accessibility, and (iii) possible interactions of iPsiR with endogenous transcriptional cofactors or repressor complexes. This phenomenon was observed in other control systems or genetic switches [11, 28]. Future achievement of uniform performance across diverse cell types will likely require tailored strategies, such as promoter engineering or delivery optimization [35, 38, 39].

To evaluate the long‐term gene of interest (GOI) regulation and potential therapeutic efficacy of the PURE system, we co‐transfected the PURE components: iPsiR (pQL450; ITR‐P_hEF1α_‐KRAB‐PsiR_T135N;V134S_‐pA::P_mPGK_‐PuroR‐pA‐ITR) and P_PsiR3_‐driven SEAP and insulin (pQL451; ITR‐P_PsiR3_‐SEAP‐P2A‐mINS‐pA::P_mPGK_‐ZeoR‐P2A‐EGFP‐pA‐ITR) and generated a stable monoclonal PURE‐transgenic HEK‐293T cell line HEK_PURE‐SEAP_ by Sleeping Beauty transposase (SB) system [29], which constitutively and stably expressed iPsiR and P_PsiR3_‐driven SEAP. Monoclonal cell line no. 19 showed the highest induction fold of the SEAP reporter gene (∼58.4‐fold) with low leakiness (Figure 2C).

We next characterized the stable HEK_PURE‐SEAP_ cell line. Induction with psicose resulted in dose‐dependent SEAP expression sustained for up to 6 days (Figure 2D). Notably, it took at least 6 h to activate the PURE system. Moreover, a significant time‐dependent increasing of SEAP expression was mediated by the PURE system (Figure S4). The cells further maintained stable output for over 13 days in continuous culture (Figure 2E). Further, we characterized the gene expression reversibility of this stable HEK_PURE‐SEAP_ cell line, which was sequentially cultured in the presence of psicose or vehicle (DMSO, dimethylsulfoxide) for 6 days, switching between conditions every 2 days, and obtained a robust ON‐OFF‐ON or OFF‐ON‐OFF expression pattern (Figure 2F), indicating that the PURE system enables reversible and tightly controlled gene activation. These results demonstrated that the PURE system can fine‐tune transgene expression in mammalian cells.

We next evaluated the impact of the psicose on the viability of mammalian cells: HEK‐293 cells were transfected with pSEAP2‐control and then exposed to different concentrations of psicose (from 0 to 20 mm). The SEAP expression showed that none of the tested psicose concentrations had a significant impact on gene expression‐based metabolic integrity (Figure S5A). Moreover, we did not observe substantially increased cytotoxicity with different concentrations of psicose exposure (Figure S5B), indicating the inertness of the system constituents.

### 2.3 Control of Transgene Expression in Mice Using the PURE System

**FIGURE 3:**
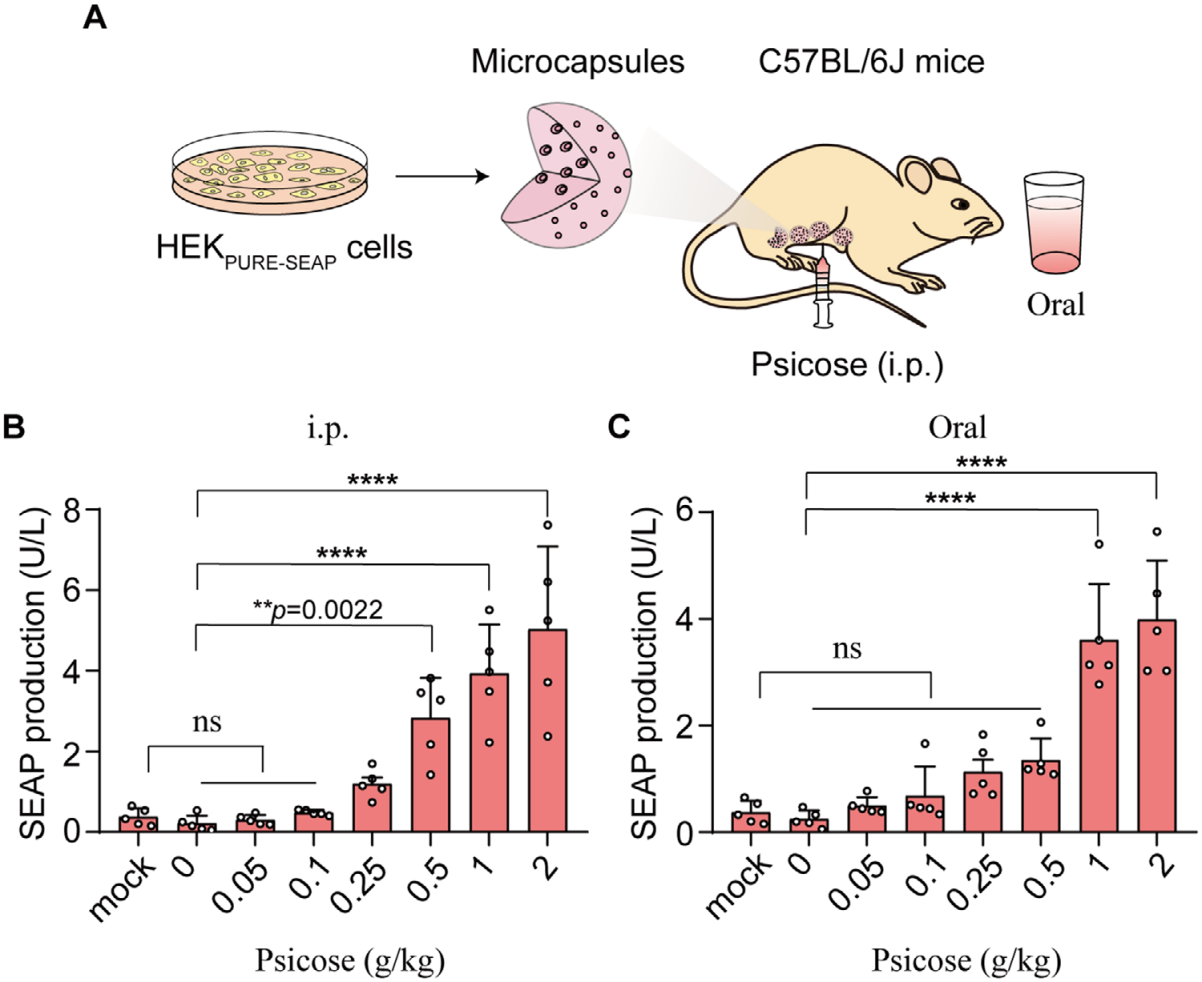
Performance of the PURE system in mice. (A) Schematic showing the mouse experimental design and procedure for assessing the PURE system in vivo. HEK_PURE‐SEAP_ cells were microencapsulated into alginate‐poly‐(l‐lysine)‐alginate beads allowing free diffusion of oxygen, nutrients, and secreted proteins across the membrane while simultaneously shielding encapsulated cells from the immune system. The microencapsulated HEK_PURE‐SEAP_ cells were implanted in the peritoneum of mice and the mice were treated via intraperitoneal injection (i.p.) or via oral administration of psicose. (B,C) Dose‐dependent psicose‐inducible SEAP expression in mice implanted with encapsulated PURE‐designer cells. 8‐week‐old male C57BL/6J mice were intraperitoneally implanted with 2.5 × 10^6^ microencapsulated HEK_PURE‐SEAP_ cells (200 cells per capsule) and received intraperitoneal injection (B) or oral administration (C) with different concentrations of psicose. After 2 days, blood samples were collected, and SEAP levels in the serum were quantified by using a chemiluminescence‐based assay kit. Data are presented as the means ± SEM, *n*  =  5 mice. *P* values in (B,C) were calculated by one‐way ANOVA with multiple comparisons. ns, not significant, ^**^p < 0.01, ^****^p < 0.0001.

Having established the basic performance characteristics of the PURE system in cells, we next conducted experiments with mice to test whether PURE could be used to control transgene expression in vivo via intraperitoneal (i.p.) injection or oral administration of psicose. Cell encapsulation is a technique that immobilizes engineered cells within a semipermeable, biocompatible membrane, allowing for controlled therapeutic protein release while shielding the cells from host immune rejection [30], which has been employed for implantable living cell systems in both preclinical and clinical drug delivery settings [31]. So, the designer cells (HEK_PURE‐SEAP_) equipped with this PURE system were encapsulated within alginate–poly(l‐lysine)–alginate microbeads. The encapsulated cells were then implanted into the peritoneal cavity of mice (Figure 3A).

Analysis of blood samples revealed significantly increased SEAP levels in mice through either i.p. injection or oral administration of psicose (Figure 3B,C), indicating successful in vivo activation of the transgene. Moreover, SEAP expression showed a dose‐dependent increase in response to escalating concentrations of psicose (0 to 2 g/kg daily) (Figure 3B,C), supporting the tunability of the PURE system in mice. Notably, oral administration of psicose at a dose of 1 g/kg induced SEAP expression to 3.61 U/L in mice, reaching a plateau level, whereas only 0.24 U/L was detected in the non‐induced control group (Figure 3C).

Moreover, we observed that the PURE system exhibited different induction efficiencies in vivo depending on the route of psicose administration. Specifically, when psicose was administered via i.p. injection, the system showed a higher induction efficiency via i.p. injection compared to oral administration (Figure 3B,C). Such differences in response effects between injection and oral administration could probably be two pharmacokinetic factors: (i) absorption rate and bioavailability: psicose is rapidly absorbed into the bloodstream and bypassed the gastrointestinal tract and first‐pass metabolism by i.p. injection, leading to higher bioavailability and faster onset of action. (ii) gastrointestinal transit time: psicose is required to traverse the digestive system by oral administration, which can delay absorption and reduce the amount of systemic circulation.

Oral delivery of the inducer offers a more patient‐friendly and compliant route for long‐term therapeutic applications compared to i.p. injection [11]. Those results demonstrated that introducing PURE designer cells into mice can achieve high expression of a GOI into the circulatory system in response to a psicose inducer taken orally by mice. So, we employed oral administration in subsequent in vivo experiments.

To evaluate the safety of oral psicose administration, healthy wild‐type C57BL/6 mice were randomly divided into two groups and treated daily with either psicose (1 g/kg) or an equal volume of PBS for 2 weeks. Histological examination (H&E) staining revealed no detectable tissue damage in psicose‐treated mice compared with PBS controls (Figure S6). Furthermore, inflammatory cytokine levels did not differ significantly between the two groups (Figure S7). These results indicate that oral psicose administration is well tolerated and supports its safety as an inducer in vivo.

To evaluate the safety of microencapsulated HEK_PURE‐SEAP_ cells transplantation. C57BL/6 mice were implanted intraperitoneally with microencapsulated HEK_PURE‐SEAP_ cells for 2 weeks, followed by H&E staining of major organs, including the heart, liver, spleen, lung, and kidney. No detectable tissue damage or inflammatory lesions were observed in HEK_PURE‐SEAP_ cells‐treated mice compared with wild‐type mice (Figure S8). These results indicate that transplantation of HEK_PURE‐SEAP_ cells—stably engineered using the SB transposon system—does not cause detectable systemic toxicity or histopathological abnormalities, supporting the biosafety and biocompatibility of the PURE system in vivo.

### 2.4 Designer Cells Containing the PURE System for Diabetes Therapy in a T1D Mouse Model

Poor treatment adherence is a major issue among diabetes patients who require daily insulin self‐injection, putting them at risk for inadequate glycaemic control [32]. Several food‐ or beverage‐derived compounds, including protocatechuic acid and non‐natural amino acids, have been employed as oral inducers for regulating insulin expression in diabetic animal models [11, 33]. Given that psicose is commonly used as a sugar substitute for glucose or sucrose, and considering our data demonstrating high sensitivity of the designer cells (HEK_PURE‐SEAP_) to psicose (Figure 3C), we explored the feasibility of using psicose as a food additive to trigger therapeutic gene expression.

Customized diabetic‐friendly products—such as sugar‐free beverages and low‐calorie foods—are widely accepted among diabetes patients and readily available through the food retail supply chain [34]. We therefore envisioned a therapeutic application in which “psicose cola” served as an orally administered trigger for insulin expression of the implanted PURE designer cells. Then we formulated psicose cola by supplementing commercially available sugar‐free cola with psicose.

Commercial sugar‐free cola contains multiple components (e.g., caramel color, phosphoric acid, preservatives, and flavoring agents); So, we first sought to exclude the possibility that these additional constituents could activate the PURE system. Streptozotocin (STZ)‐induced T1D mice were randomly allocated into four experimental groups: G1, STZ‐induced T1D control mice (STZ); G2, STZ‐induced T1D mice implanted with microencapsulated HEK_PURE‐SEAP‐2A‐mINS_ cells and exposed to cola (HEK_PURE‐SEAP‐2A‐mINS_ + cola); G3, STZ‐induced T1D mice implanted with microencapsulated HEK_PURE‐SEAP‐2A‐mINS_ cells without cola exposure (HEK_PURE‐SEAP‐2A‐mINS_—psicose cola); and G4, STZ‐induced T1D mice implanted with microencapsulated HEK_PURE‐SEAP‐2A‐mINS_ cells and exposed to psicose cola (HEK_PURE‐SEAP‐2A‐mINS_ + psicose cola). We found that T1D mice implanted with HEK_PURE‐SEAP‐2A‐mINS_ cells and exposed to psicose cola (G4) had higher blood insulin levels, whereas insulin levels in the G2 group were not different from that in the G1 and G3 groups (Figure S9). These findings indicate that the PURE system is highly specific for psicose added into cola, not for cola.

To determine the optimal dosing frequency, we first investigated the in vivo kinetic parameters of insulin expression activated by the PURE system upon psicose. the STZ‐induced T1D mice were implanted with HEK_PURE‐SEAP‐2A‐mINS_ cells and received a single oral administration of psicose cola, and insulin levels were measured at different time points (0–36 h). The results showed that insulin levels increased to a peak at approximately 18 h post‐administration. Thereafter, insulin levels gradually decreased but remained markedly elevated above baseline until 24 h (Figure S10). Based on these findings, the dosing interval was set at every 24 h in our study.

**FIGURE 4:**
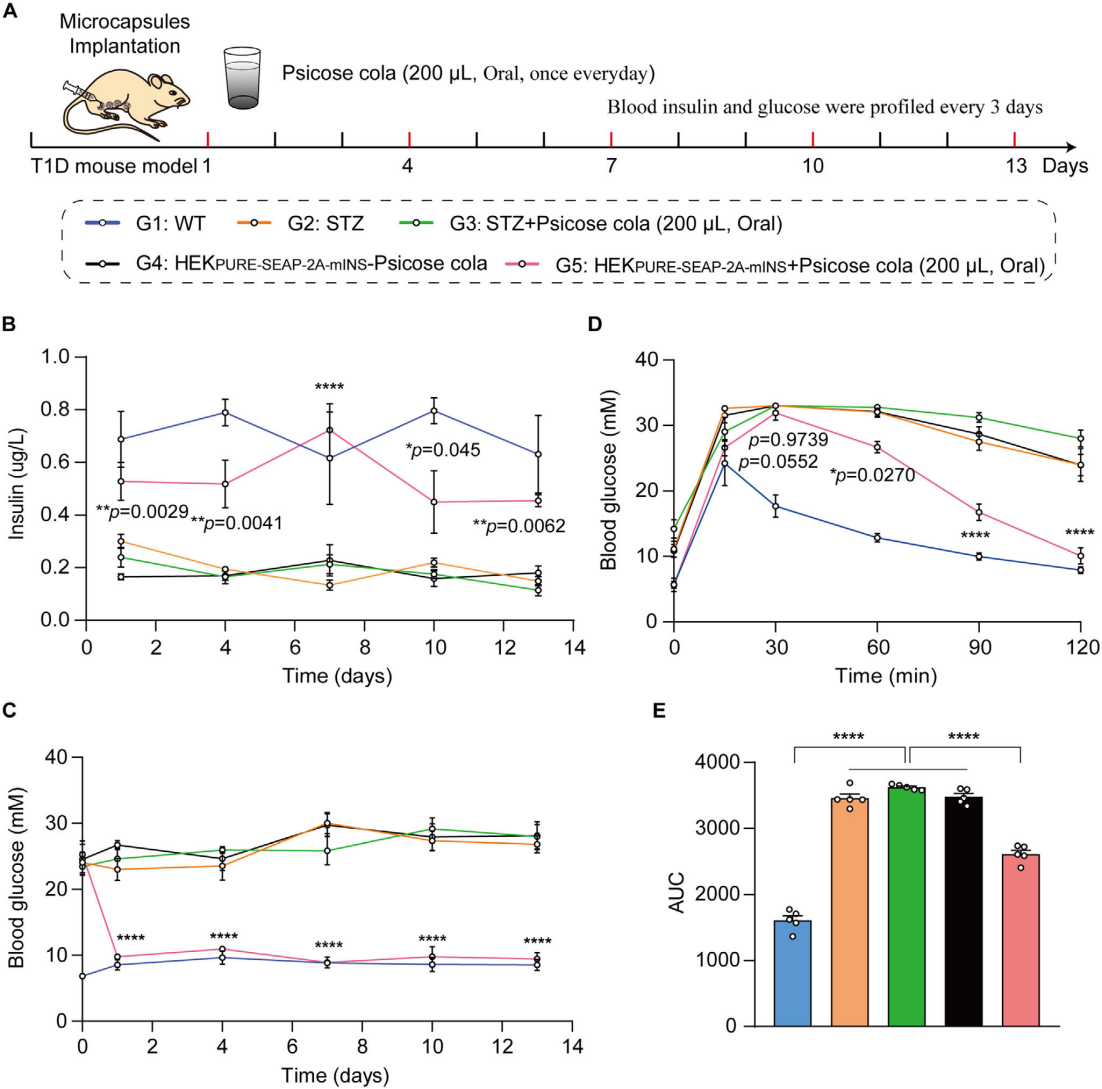
Therapeutic efficacy of PURE designer cells in T1D mice by drinking psicose cola. (A) Schematic showing the mouse experimental design and procedure for HEK_PURE‐SEAP‐2A‐mINS_ cell therapy in T1D mouse model by drinking psicose cola (oral administration).The wild‐type (WT) mice and streptozotocin (STZ)‐induced type 1 diabetic mice were divided into five experimental groups: G1, WT; G2, STZ; G3, STZ + psicose cola; G4, HEK_PURE‐SEAP‐2A‐mINS_—psicose; and G5, HEK_PURE‐SEAP‐2A‐mINS_ + psicose cola (A). Mice in groups G3 and G5 received daily oral administration of 200 µL psicose cola (corresponding to 1 g psicose per kg body weight). Serum insulin level (B) and blood glucose (C) were profiled every 3 days. Blood glucose concentration (D) and the corresponding area (E) under curve of the intraperitoneal glucose tolerance was analyzed 72 h after cell implantation. Data are presented as the means ± SEM; *n*  =  5 mice. *P* values in (B–D) were calculated using a two‐tailed unpaired *t*‐test. *P* values in (E) were calculated by one‐way ANOVA with multiple comparisons. AUC, area under curve. ns, not significant, ^*^p < 0.05, ^**^p < 0.01, ^****^p < 0.0001.

To evaluate the long‐term efficacy of psicose cola–mediated insulin expression of the PURE system for blood glucose control in T1D mice, STZ‐induced T1D mice were randomly divided into four experimental groups mice, while wild‐type (WT) mice as control: G1, WT group; G2, STZ‐induced T1D control mice (STZ) group; G3, STZ‐induced T1D control mice exposed to psicose cola (STZ + psicose cola) group; G4, STZ‐induced T1D mice implanted with microencapsulated HEK_PURE‐SEAP‐2A‐mINS_ without psicose cola exposure (HEK_PURE‐SEAP‐2A‐mINS_—psicose cola) group; and G5, STZ‐induced T1D mice implanted with microencapsulated HEK_PURE‐SEAP‐2A‐mINS_ with psicose cola exposure (HEK_PURE‐SEAP‐2A‐mINS_ + psicose cola) group (Figure 4A). T1D mice implanted with HEK_PURE‐SEAP‐2A‐mINS_ cells and exposed to psicose cola (G5) showed higher blood insulin levels (Figure 4B) and lower fasting glucose levels (Figure 4C) over a 13 day period compared to T1D mice implanted with HEK_PURE‐SEAP‐2A‐mINS_ cells without psicose cola exposure (G4), and STZ‐induced T1D control mice with or without psicose cola exposure (G2, G3).

Moreover, Blood glucose and insulin levels in G3 were indistinguishable from those in G2 and G1 (Figure 4B), indicating that psicose cola alone did not affect glucose metabolism. Specifically, blood glucose levels decreased from an initial 23 mm to approximately 10 mm, comparable to normal glycaemic levels observed in WT mice G1 (Figure 4C). Finally, an intraperitoneal glucose tolerance test (IPGTT) was performed 3 days after implantation of the PURE designer cells. G5 mice showed significantly improved glucose homeostasis and reduced postprandial glucose excursions compared to other control group mice (G2, G3, and G4) (Figure 4D,E). Collectively, these results indicate that the PURE system can safely regulate insulin expression to achieve the desired blood glucose levels in T1D mice.

### 2.5 Control of TSLP Expression by the Designer Cells Containing the PURE System in HFD‐Induced Obesity Model Mice

**FIGURE 5:**
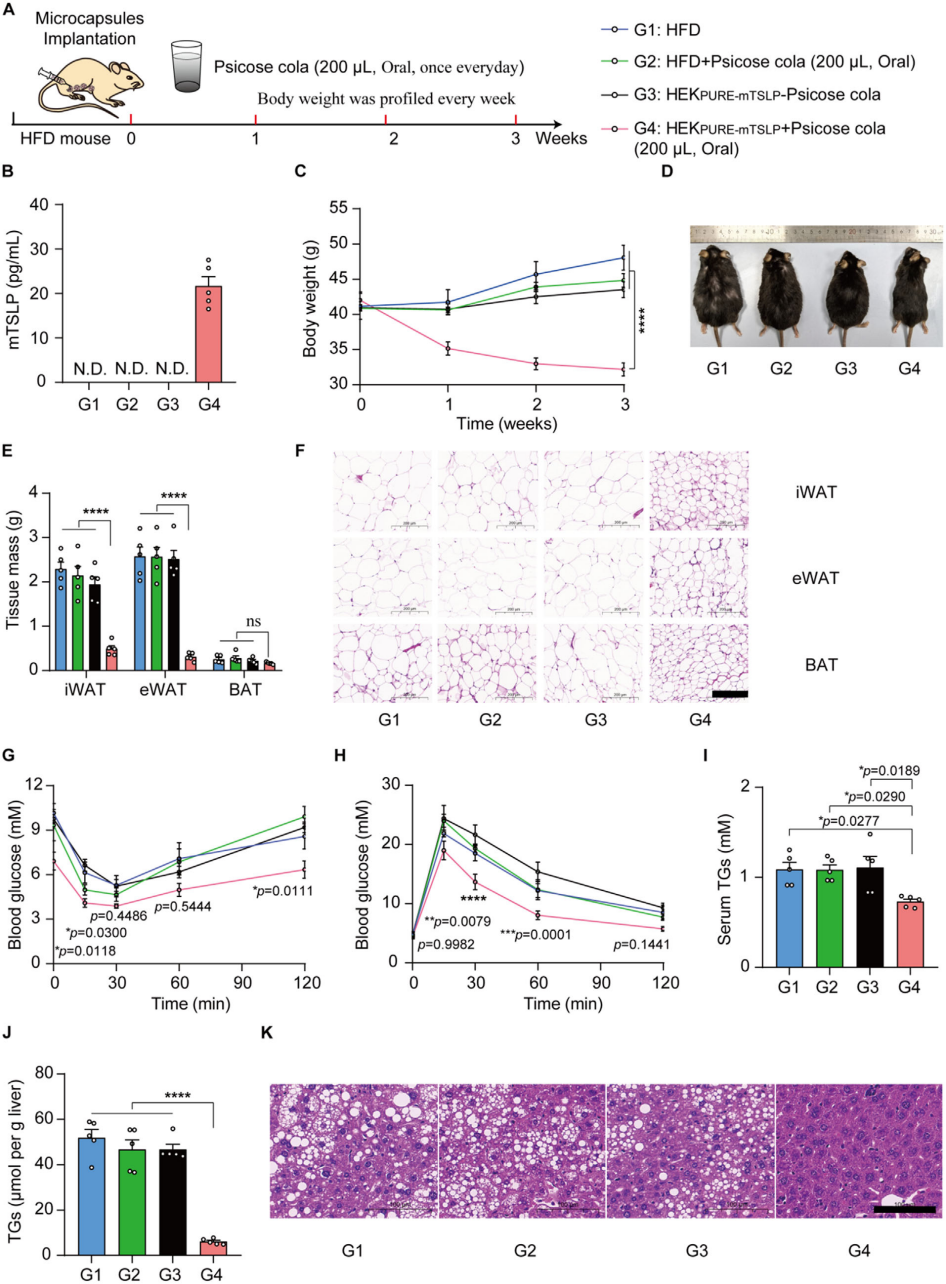
Therapeutic efficacy of PURE designer cells in HFD mice by drinking psicose cola. (A) Schematic showing the mouse experimental design and procedure for HEK_PURE‐mTSLP_ cell therapy in the HFD mouse model. HFD‐fed mice were divided into four groups: G1, HFD; G2, psicose cola; G3, HEK_PURE‐mTSLP_—psicose cola; and G4, HEK_PURE‐mTSLP_ + psicose cola. Mice in groups G2 and G4 received daily oral administration of 200 µL psicose cola (corresponding to 1 g psicose per kg body weight). After 1 day, the levels of mTSLP expression (B) in serum were quantified using a mouse TSLP ELISA kit. N.D., Not detected. (C) Mice were weighed weekly. (D) Representative images of mice after 3 weeks of treatment. (E) Analysis of adipose tissue (iWAT, eWAT, and BAT) weights in different treated HFD mice. (F) Representative H&E staining of the adipose tissues. Scale bars = 200 µm. Analysis of metabolic parameters: (G) IPGTT. (H) ITT. (I) Serum TG. (J) Liver TG. (K) Representative H&E staining of the liver. Scale bars = 100 µm. Data are presented as the means ± SEM; *n*  =  5 mice. *P* values in (C,E,I,J) were calculated by one‐way ANOVA with multiple comparisons. *P* values in (G,H) were calculated using a two‐tailed unpaired *t*‐test. ns, not significant, ^*^p < 0.05, ^**^p < 0.01, ^***^p < 0.001, ^****^p < 0.0001.

We next tested the applicability of the PURE system to control the expression of mTSLP using psicose cola as a trigger for implanted HEK_PURE_‐designer cells in HFD‐induced obesity model mice. mTSLP has been recently reported to protect against obesity and obesity‐related complications [35]. We also engineered a stable cell line to induce mTSLP expression in response to psicose cola using SB transposon‐based vectors and implanted PURE engineered cells into HFD‐induced obesity model mice. HFD‐fed mice were divided into four groups: G1, HFD group; G2, psicose cola alone group; G3, HFD‐fed mice implanted with microencapsulated HEK_PURE‐mTSLP_ without psicose cola exposure (HEK_PURE‐mTSLP_‐psicose cola) group; and G4, HFD‐fed mice implanted with microencapsulated HEK_PURE‐mTSLP_ with psicose cola exposure (HFD‐fed mice implanted with microencapsulated HEK_PURE + mTSLP_) group. Mice in groups G2 and G4 received daily oral administration of 200 µL psicose cola (corresponding to 1 g psicose per kg body weight) (Figure 5A).

Twenty‐four hours post‐induction, a marked increase in serum mTSLP levels was observed in G4 mice, whereas no detectable mTSLP was found in any of the three control groups (Figure 5B). Over the course of a 3 week treatment period, HFD‐fed mice implanted with microencapsulated HEK_PURE‐mTSLP_ with psicose cola exposure (G4) mice had significantly reduced body weight compared to the HEK_PURE‐mTSLP_‐psicose cola group, psicose cola alone group, and the untreated HFD control mice (Figure 5C,D). After 3 weeks of treatment in the HFD model mice, the G4 mice displayed significantly decreased weights of adipose tissues (including subcutaneous inguinal white adipose tissue (iWAT) and epididymal white adipose tissue (eWAT)) compared to the other three control groups. The brown adipose tissue (BAT) was not changed among all the groups (Figure 5E). Moreover, H&E staining revealed that the G4 mice exhibited significantly smaller adipocyte sizes in adipose tissues (iWAT, eWAT, and BAT) compared to the other three control groups (Figure 5F).

A battery of metabolic tests, including an IPGTT (Figure 5G), an insulin tolerance test (ITT) (Figure 5H), as well as measuring serum and liver triglycerides (TGs) (Figure 5I,J), consistently showed significant decreases in the G4 group compared to the other three controls. In addition, HE staining showed that the G4 group had significantly smaller fat droplets, indicating a reduced severity of hepatic steatosis in the liver (Figure 5K). These results demonstrated the feasibility of beverage‐compatible induction of therapeutic protein expression in vivo and suggested a potential strategy for obesity intervention through engineered living cell therapeutics.

## 3 Discussion

Cells endowed with synthetic gene circuits can control timing and dosage of therapeutic activities in response to specific inducer, such as psicose, thereby representing a powerful new weapon in the fight against disease. In this study, we have developed a psicose‐controlled transgene expression system (PURE system) based on the mutated PsiR to preciously control transgene expression with a dose‐dependent manner and reversible regulation properties. Further, the PURE system is highly specific to psicose, being insensitive to other sugars and structurally similar molecules. As a proof‐of‐concept, we demonstrated that microencapsulated designer cells can be activated by drinking psicose cola to regulate therapeutic protein expression in vivo, achieving glycaemic control in diabetic mice and body weight reduction in obese mice.

Some therapies will require the long‐term delivery of trigger molecules; the exogenous stimulus should be carefully selected to ensure no toxicity or side effects and compatibility with long‐term application in humans. Previous studies have developed the SWEET system for the treatment of T1D mice using xylose as an inducer [14]. However, xylose is easily metabolized by gut microbes to produce flatulence, leading to side effects of long‐term treatment [36]. We chose to formulate psicose as an oral beverage to potentially improve patient compliance in the treatment of diseases, given its safety designation as GRAS in 2011. Psicose offers several advantages over other sweeteners when it was used to induce gene expression: it is natural, inexpensive, and has a short blood half‐life. Therefore, it is safer than artificial sweeteners and compatible with the needs of patients. In addition, psicose supplemented in diet appears to have beneficial effects on lipid metabolism and weight management [37].

Our study demonstrated how the PURE system can be seamlessly integrated with the convenient delivery method of a psicose beverage, providing a novel approach for food‐based cell therapies for treating diseases such as obesity and other metabolic diseases. Moreover, this strategy could be extended to induce the expression of urate oxidase for gout treatment [38] or parathyroid hormone (PTH) for hyperparathyroidism [39].

While the PURE system has effectively controlled the condition of mice modeling diseases, there remains room for improving the sensitivity of this system. For example, channel proteins will be constructed to pump psicose cola into designer cells to increase the sensitivity of this system. Collectively, we expect that the PURE system will accelerate the progression of cell and gene therapies toward future clinical applications.

## 4 Conclusion

In this study, we developed a natural sweetener–inducible transgene expression system (PURE) based on the *Agrobacterium tumefaciens–*derived transcriptional repressor PsiR. Using bioinformatics‐guided analysis and rational mutagenesis, we engineered a more sensitive variant of PsiR (PsiR_T135N;V134S_). As a proof‐of‐concept, the PURE system was shown to regulate insulin expression and effectively lower blood glucose levels in a T1D mouse model, as well as to induce the expression of an anti‐obesity therapeutic protein (mTSLP), leading to body weight reduction in obese mice following administration of a psicose‐containing “cola.”

## 5 Experimental Section/Methods

### 5.1 Ethical Statement

The experiments involving animals were performed according to the protocol approved by the East China Normal University (ECNU) Animal Care and Use Committee and were in direct accordance with the Ministry of Science and Technology of the People's Republic of China on Animal Care Guidelines. The protocol was approved by the ECNU Animal Care and Use Committee (protocol ID: AR2023‐128). All mice were euthanized after the termination of the experiments.

### 5.2 Plasmid Construction

Construction details of the plasmids are provided in Table S1. The DNA sequences for the PURE system components in this study are listed in Table S4. The plasmids were constructed by Gibson assembly according to the manufacturer's instruction (MultiS One Step Cloning Kit, Catalog no. C113‐01, Vazyme) and the sequences were confirmed by DNA sequencing (Shanghai Saiheng Biotechnology).

### 5.3 Chemicals

For cell culture incubation, psicose (Catalog no. M19495, MERYER), allose (Catalog no. A606673‐0100, Sangon Biotech), fructose (Catalog no. A600213‐0500, Sangon Biotech), galactose (Catalog no. A600215‐0025, Sangon Biotech), mannose (Catalog no. A600554‐0025, Sangon Biotech), ribose (Catalog no. A610475‐0025, Sangon Biotech), xylose (Catalog no. A600998‐0100, Sangon Biotech), arabinose (Catalog no. A600071‐0025, Sangon Biotech) were prepared in 2 m stock solutions in sterilized ddH_2_O, stored at 4 ℃, and diluted to the final working concentrations using DMEM medium. For animal experiments, psicose was dissolved in sterilized ddH_2_O or sugar‐free cola (Catalog no. 6928804015157, Shanghai Shenmei Beverage and Food Co., Ltd) at a 250 mg/mL stock solution, and then diluted to the final working concentrations using saline solution (0.9% NaCl).

### 5.4 Cell Culture and Transfection

Human embryonic kidney cells (HEK‐293T, RRID: CVCL_1926, Catalog no. CRL‐11268, ATCC), telomerase‐immortalized human mesenchymal stem cells (hMSC‐TERT, which was obtained from Professor Dr. Martin Fussenegger Department of Biosystems Science and Engineering, ETH Zurich.), human cervical adenocarcinoma cells (HeLa, RRID: CVCL_0030, Catalog no. CCL‐2, ATCC) and baby hamster kidney‐21 cells (BHK‐21, RRID: CVCL_1915, Catalog no. CCL‐10, ATCC) were cultured in Dulbecco's modified Eagle's medium (DMEM, Catalog no. 12100061, Gibco) supplemented with 10% (v/v) fetal bovine serum (Catalog no. FBSSA500‐S, AusGeneX), and 1% (v/v) penicillin/streptomycin solution (Catalog. no. ST488‐1/ST488‐2, Beyotime). The melanoma cells (B16F10, RRID: CVCL_0159, Catalog no. NM‐S31A‐TG05, Shanghai Model Organisms Center) were cultured in RPMI‐1640 (Catalog no. 8122663, Gibco) containing 10% FBS (Catalog no. FBSSA500‐S, AusGeneX) and 1% (v/v) penicillin/streptomycin solution. All the cell lines were cultured at 37°C in a humidified atmosphere containing 5% CO_2_, and were regularly tested for the absence of Mycoplasma and bacterial contamination. The concentration and viability of the cell lines were evaluated using a Countess II Automated cell counter (AMEP4746, Life Technologies). All cell lines were confirmed to have mycoplasma contamination.

All cells except for HeLa and B16F10 were transfected using an optimized polyethyleneimine (PEI)‐based protocol. Briefly, 5 × 10^4^ cells per well were plated into a 24‐well plate and cultured for 18 h. The cells were subsequently co‐transfected with corresponding plasmid mixtures for 6 h with 50 µL PEI (Catalog no. 24765, Polysciences; molecular weight 40 000, stock solution 1 mg/mL in ddH_2_O; A mass ratio of PEI and DNA is 3:1. For the HeLa and B16F10 cells, 5 × 10^4^ cells per well were plated into a 24‐well plate, cultured for 18 h, and co‐transfected with corresponding plasmid mixtures using Lipofectamine 3000 (Catalog. no. L3000015, Thermo Fisher Scientific) according to the manufacturer's instructions.

### 5.5 Structure Prediction and Molecular Docking of PsiR

The PsiR structure was predicted using RosettaFold with default parameters. Templates were automatically selected based on multiple sequence alignment (MSA), and the quality of the predicted structural model was primarily evaluated using the GDT metric, which performed a structural agreement analysis on a set of partially threaded models derived from the top‐ranked alignments of each independent method. Molecular docking was performed with a grid box of 51.5 × 51.5 × 51.5 Å centered at the geometric center of PsiR. The docking performance was evaluated based on the binding affinity between PsiR and its ligand, with ten independent runs conducted for each docking simulation.

### 5.6 MTT Assay

HEK‐293T cells were seeded (1 × 10^4^ cells/per well) in a 96‐well plate and incubated with various concentrations of psicose for 48 h. 10 µL MTT solution (5 mg/mL in PBS, Catalog. no. A600799, Sangon Biotech) was then added to each well and incubated for 4 h at 37°C. The reaction was terminated with 100 µL of a solubilization solution containing 5% isobutanol, 10% sodium dodecyl sulfate, and 0.012 mol/L HCl. Plates were read with the Synergy H1 hybrid multi‐mode microplate reader (BioTek Instruments) at 570 nm after the complete solubilization of purple formazan crystals.

### 5.7 SEAP Assay

A p‐nitrophenylphosphate–based light absorbance time course assay was used to quantify the production of human placental SEAP. Briefly, 100 µL cell culture supernatants were heat‐inactivated at 65°C for 30 min. 120 µL of substrate solution [100 µL of 2 × SEAP buffer (pH 9.8) containing 20 mm L‐homoarginine hydrochloride (Catalog no. A602842, Sangon Biotech), 1 mm MgCl_2_ (Catalog no. A610328, Sangon Biotech), 21% (w/w) diethanolamine (Catalog no. A600162, Sangon Biotech), and 20 µL of p‐NPP substrate solution (Catalog no. 333338‐18‐4, Sangon Biotech) containing 120 mm p‐nitrophenylphosphate] was added to 80 µL heat‐inactivated supernatants. The time course of the absorbance at 405 nm was measured by the Synergy H1 hybrid multi‐mode microplate reader (BioTek Instruments) with Gen5 software (version: 2.04).

### 5.8 Generation of Stable Cell Lines

The HEK_PURE‐SEAP‐P2A‐mINS_ cell line, transgenic for psicose‐triggered insulin and SEAP expression, was constructed by co‐transfecting 5 × 10^4^ HEK‐293T cells with 100 ng of pQL451 (ITR‐P_PsiR3_‐SEAP‐P2A‐mINS‐pA::P_mPGK_‐ZeoR‐pA‐ITR), 100 ng of pQL450 (ITR‐P_hEF1α_‐KRAB‐PsiR_T135N;V134S_‐pA::P_mPGK_‐PuroR‐pA‐ITR), and 20 ng of Sleeping Beauty transposase expression vector pCMV‐T7‐SB100 (P_hCMV_‐SB100X‐pA). The HEK_PURE‐SEAP‐P2A‐mTSLP_ cell line, transgenic for psicose‐triggered mTSLP and SEAP expression, was constructed by co‐transfecting 5 × 10^4^ HEK‐293T cells with 100 ng of pQL452 (ITR‐P_PsiR3_‐mTSLP‐pA::P_mPGK_‐ZeoR‐pA‐ITR), 100 ng of pQL450 (ITR‐P_hEF1α_‐KRAB‐PsiR_T135N;V134S_‐pA::P_mPGK_‐PuroR‐pA‐ITR), and 20 ng of Sleeping Beauty transposase expression vector pCMV‐T7‐SB100 (P_hCMV_‐SB100X‐pA). After cultivation with 1 µg/mL puromycin (Catalog no. A1113803, Thermo Fisher Scientific) and 100 µg/mL zeocin (Catalog no. R25001, Thermo Fisher Scientific) for 2 weeks, the monoclonal cell lines were selected and identified.

### 5.9 Long‐Term SEAP Expression Kinetics and Reversibility of Psicose‐Controlled HEK_PURE‐SEAP_ Stable Cells

To test long‐term SEAP production kinetics of HEK_PURE‐SEAP_ stable cells, 1 × 10^5^ cells were seeded in a 10 cm‐dish and incubated with or without 5 mm psicose for 13 days. The culture medium was replaced with fresh medium every 48 h, and SEAP production was profiled every 24 h.

To evaluate the reversibility of HEK_PURE‐SEAP_‐mediated SEAP production. 5 × 10^4^ cells were seeded in a 24‐well plate and cultivated for 6 days while altering psicose concentrations from 0 to 2.5 mm. Cell density was adjusted to 5 × 10^4^ every 48 h, and SEAP production was profiled every 12 h.

### 5.10 Animals

All animals were approved by the Institutional Animal Care and Use Committee of Shanghai and conducted in accordance with the National Research Council Guide for the Care and Use of Laboratory Animals. The experimental animals included 4‐ or 8‐week‐old or body weight ∼25 g C57BL/6 male mice. Mice were reared in East China Normal University Laboratory Animal Center and kept with a standard alternating 12 h light/12 h dark cycle.

### 5.11 Construction of Mouse Model of Type 1 Diabetes (T1D)

The type 1 diabetes mouse model (T1D) was induced by streptozotocin (STZ) injection. Briefly, adult male mice (C57BL/6, with body weight ∼25 g) were fasted for 16 h and intraperitoneally injected with STZ (Catalog no. S0130, Merck; 50 mg/kg in 0.1 m citrate buffer, pH 4) every day for 5 days. 2 weeks after the initial injection, fasted mice with hyperglycemia over 16.7 mmol/L glucose were considered diabetic and used for further animal experiments.

### 5.12 Construction of Mouse Model of High‐Fat Diets (HFD)

The male mice (C57BL/6, 4‐week‐old) were fed high‐fat diets consisting of 60 kcal% fat (Catalog no. D12492, Research Diets) for 13 weeks until the body weight exceeded 40 g.

### 5.13 Microcapsule Implants in Mice

Intraperitoneal implants were prepared by encapsulating transgenic HEK‐293T cells into coherent alginate‐poly‐(l‐lysine)‐alginate beads (400 µm; 200 cells per capsule) using a B‐395 Pro encapsulator (BÜCHI Labortechnik AG) set to the following parameters: a 200 µm nozzle with a vibration frequency of 1300 Hz, a 25 mL syringe operated at a flow rate of 450 U, and 1.10 kV voltage for bead dispersion. Mice were intraperitoneally injected with 800 µL of DMEM containing 2.5 × 10^6^ microencapsulated designer cells (200 transgenic HEK‐293T cells per capsule).

For SEAP production in mice, the mice were daily intraperitoneally injected or oral administration with psicose at doses ranging from 0 to 2 g/kg. After 2 days, blood samples were collected, and SEAP levels were quantified in the serum by using a chemiluminescence‐based assay kit (Catalog no. ab133077, Abcam).

For mINS production in T1D mice, the mice were received a daily oral administration of 200 µL of psicose cola containing a final psicose dose equivalent to 1 g/kg body weight. Blood insulin was profiled every 3 days using a mouse insulin ELISA kit (Catalog no. 10‐1247‐01, Mercodia), fasting blood glucose was profiled after 4 h of food restriction every 3 days using a blood glucose meter (Accu‐Check Instant, Roche).

To exclude the possibility that other components in commercial sugar‐free cola could activate the PURE system, mice received a daily oral administration of 200 µL of commercial sugar‐free cola, and blood insulin levels were measured 24 h later using a mouse insulin ELISA kit.

For mTSLP production in HFD mice, the mice were received a daily oral administration of 200 µL of psicose cola, containing a final psicose dose equivalent to 1 g/kg body weight. Blood mTSLP was profiled using a mouse TSLP ELISA kit (Catalog no. MTLP00, R&D) after psicose cola administration 1 day later. Mice were weighed weekly and the metabolic parameters were assessed, including fasting blood glucose, IPGTT, ITT, serum TG, and liver TG. The H&E staining of liver and adipose tissues was analyzed on the third week after treatment.

### 5.14 Evaluation of Oral Psicose Safety In Vivo

Healthy wild‐type C57BL/6 mice were randomly assigned to two groups and orally administered either psicose (1 g/kg) or an equal volume of PBS daily for 2 weeks. Major organs (heart, liver, spleen, lung, kidney) and intestinal tissues were collected and stained by H&E. Blood was collected, and serum TNF‐α and IL‐6 levels were quantified using mouse TNF‐α (Catalog no. EK282, Multi Science)) and IL‐6 (Catalog no. EK206, Multi Science) ELISA kit.

### 5.15 Intraperitoneal Glucose Tolerance Test (IPGTT) in Mice

Mice were fasted overnight for 16 h, and intraperitoneally injected with 10 µL 20% w/v D‐glucose dissolved in 0.85% NaCl solution per g of body weight. Blood glucose levels were measured from tail vein blood samples at 0, 15, 30, 60, 90, and 120 min after D‐glucose injection using a handheld glucometer (Exactive Easy III, MicroTech Medical). The 0 min sample was used to determine the fasting plasma glucose level. The trapezoidal rule was used to determine the area under the curve (AUC) for IPGTT.

### 5.16 Hematoxylin and Eosin (H&E) Staining

Tissues of mice were collected and fixed in 4% paraformaldehyde (Catalog no. G1101, Servicebio) overnight at room temperature. The fixed samples were gently dehydrated by immersing them in a graded series of alcohol solutions, cleaned in xylene, embedded in paraffin, and sliced into 4 µm‐thick sections with a rotary microtome (Leica RM2235, Manual Rotary Microtome). These sections were stained with an H&E Staining Kit (Catalog no. G1005, Servicebio) according to the manufacturer's instructions and were observed using an upright microscope (BX53, Olympus).

### 5.17 Statistical Analysis

Unless otherwise mentioned, all in vitro data represent means ± SD of three independent biological replicates. For the animal experiments, each treatment group consisted of randomly selected mice (*n* = 5). Comparisons between groups were performed using a two‐tailed unpaired *t*‐test or one‐way ANOVA with multiple comparisons, and the results are expressed as means ± SEM. Differences were considered statistically significant at ^*^p < 0.05, very significant at ^**^p < 0.01, extremely significant at ^***^p < 0.001, and very extremely significant at ^****^p < 0.0001. Neither animals nor samples were excluded from the study. GraphPad Prism 8.0.1 software was used for statistical analysis. n and *p* values are described in the figure legends.

## Author Contributions

F.C., H.Y., L.Q., and M.W. conceived the project. L.Q. designed the experiments, analyzed the results. L.Q. and M.W. wrote the manuscript. L.Q., Z.W., S.T., G.Y., X.Q., L.N., X.M., T.Y., X.L., D.K., Y.Z., N.G., Y.F., and J.T. performed the experimental work. L.Q. designed, analyzed, and interpreted the experiments. All authors edited and approved the manuscript.

## Funding

This work was financially supported by grants from the Young Scientists Fund of the National Natural Science Foundation of China (no. 3250120102), the Postdoctoral Fellowship Program, and China Postdoctoral Science Foundation (no. BX20250121, 2025M772716) to L.Q. This work was also partially supported by the National Natural Science Foundation of China (no. 32171414), the Natural Science Foundation of Shanghai (no. 23ZR1419500), and the Natural Science Foundation of Chongqing, China (no. CSTB2022NSCQ‐MSX0461) to M.W., the Young Scientists Fund of the National Natural Science Foundation of China (no. 32401213) to G.Y., School of Medicine, the Noncommunicable Chronic Diseases‐National Science and Technology Major Project (no. 2023ZD0501300), the Science and Technology Commission of Shanghai Municipality (no. 23HC1410100, the Sixth Cycle Key Discipline Funding from Tongji Hospital, Tongji University (no. ZDTS24‐RX, GJPY2337, GJPY2402) to F.C, the National Natural Science Foundation of China (no. 32250010, 32261160373, and 32430064), 25HC2830300), and the Fundamental Research Funds for the Central Universities to H.Y. H.Y. is a SANS Exploration Scholar.

## Conflicts of Interest

The authors declare no conflicts of interest. L.Q., F.C., and S.T. are inventors on a patent application (Chinese patent application no. 202511567293.6) filed by Tongji Hospital, School of Medicine, Tongji University, which covers the PURE system.

## Supporting information




**Supporting File**: advs73596‐sup‐0001‐SuppMat.pdf.

## Data Availability

(All data associated with this study are present in the paper or the Supplementary Information. All genetic components related to this paper are available with a material transfer agreement and can be requested from L.Q. (qiaoll@tongji.edu.cn).
