# Supplementary material for: A Natural Sweetener‐inducible Genetic Switch Controls Therapeutic Protein Expression in Mammals
Source: Adv Sci (Weinh). 2026 Jan 4;13(18):e14226. doi: 10.1002/advs.202514226 (PMC13042381; doi:10.1002/advs.202514226)
Supplement: Supplementary file 1 — Supporting File: advs73596‐sup‐0001‐SuppMat.pdf. [file ADVS-13-e14226-s001.pdf]

Supplementary Information

A Natural Sweetener-inducible Genetic Switch Controls Therapeutic Protein Expression in Mammals

Longliang Qiao, Zhihao Wang, Shasha Tang, Yuan Fang, Guiling Yu, Xiaoting Qiu, Lingxue Niu, Tao Yan, Xingwan Liu, Xiaoding Ma, Deqiang Kong, Yang Zhou, Ningzi Guan, Jinzhong Tian, Meiyan Wang*, Haifeng Ye*, Fengfeng Cai**

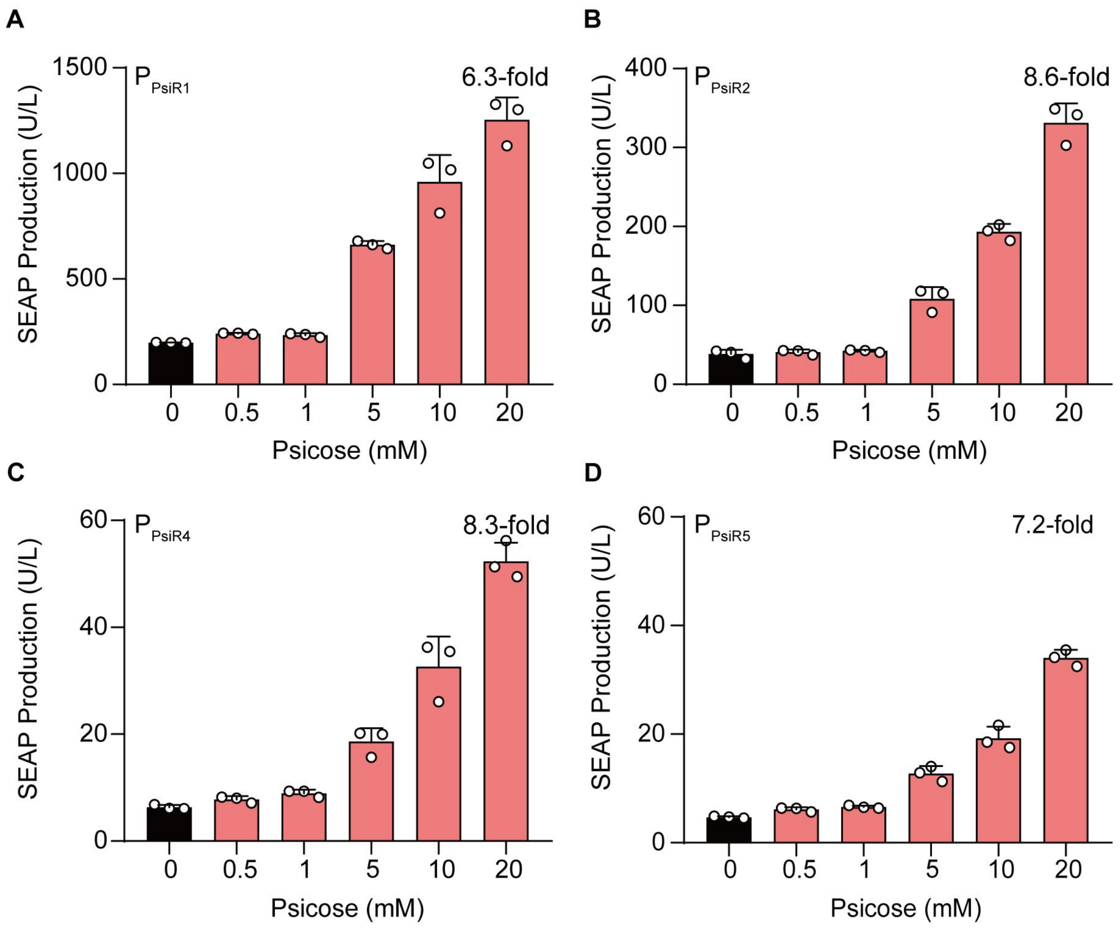

Supplementary Figure 1 Performance optimization of the PURE system by alternating different copy PsiR binding sequence (PsiO) in HEK-293T cells. HEK-293T cells (5×10^4) were co-transfected with 100 ng pQL172 ($P_{hEF1\alpha}$-KRAB-PsiR-pA) and 100 ng of different psicose-inducible SEAP expression vectors: pQL162 [P_{PsiR1}-SEAP-pA; P_{PsiR1}, P_{hCMV}-$(PsiO)_1$] (**A**), pQL163 [P_{PsiR2}-SEAP-pA; P_{PsiR4}, P_{hCMV}-$(PsiO)_2$] (**B**), pQL165 [P_{PsiR4}-SEAP-pA; P_{PsiR4}, P_{hCMV}-$(PsiO)_4$] (**C**), or pQL166 [P_{PsiR5}-SEAP-pA; P_{PsiR5}, P_{hCMV}-$(PsiO)_5$] (**D**) and cultivated in medium containing various psicose concentrations for 48 hours before SEAP in the culture supernatants was profiled. All data are presented as means ± SD; n = 3 independent experiments. Detailed descriptions of the genetic constructs and transfection mixtures are provided in Supplementary Tables 1 and 3.

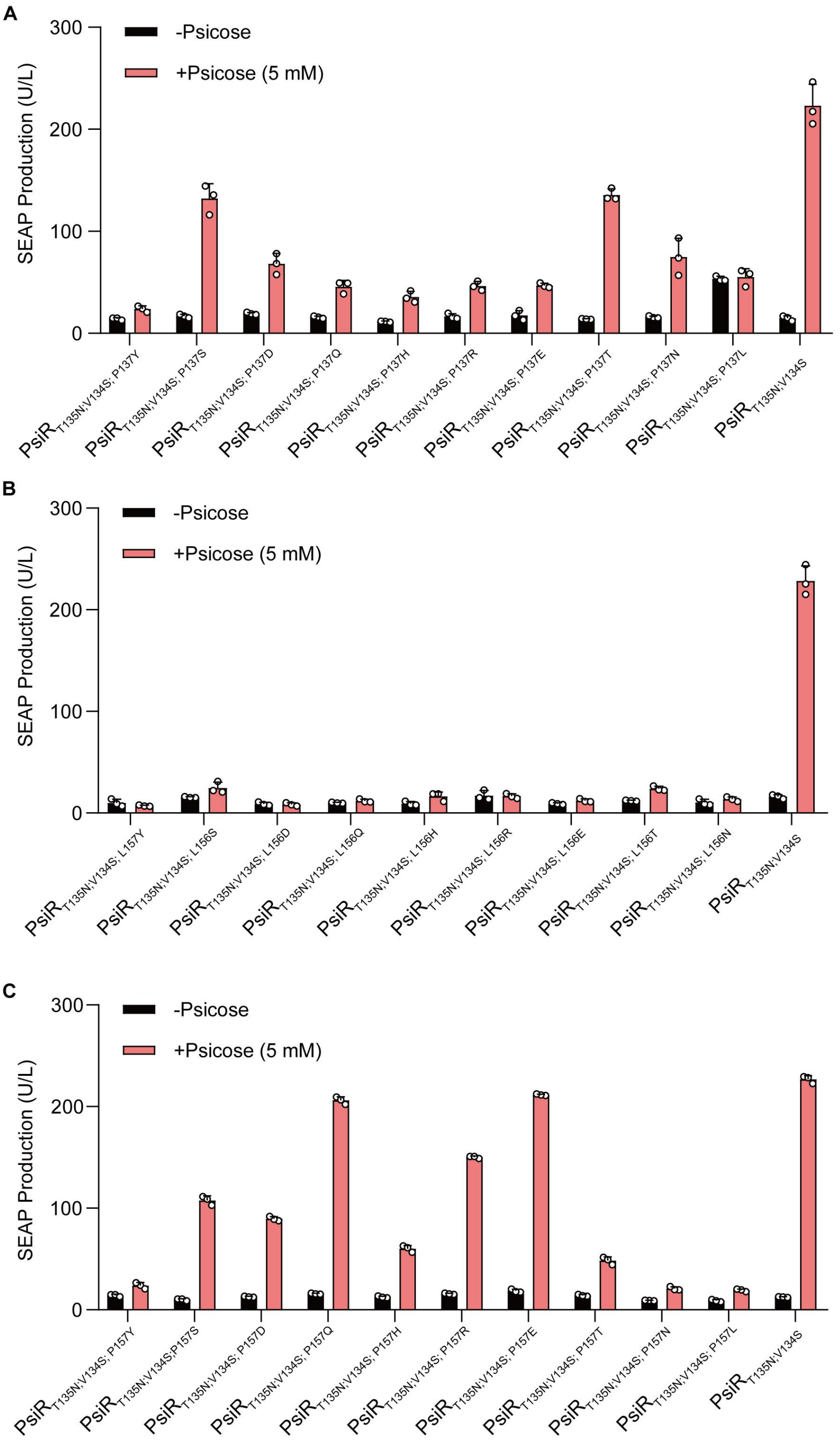

Supplementary Figure 2 Performance of different mutants of PsiR$_{T135N;V134S}$. A-C, HEK-293T cells (5 × 10^4) were co-transfected with 100 ng pQL163, 100 ng of indicated mutants of KRAB-PsiR$_{T135N;V134S}$ expression vectors and cultivated in medium containing various psicose concentrations for 48 hours before SEAP in the culture supernatants was profiled. All data are presented as means ± SD; n = 3

independent experiments. Detailed descriptions of the genetic constructs and transfection mixtures are provided in Supplementary Tables 1 and 3.

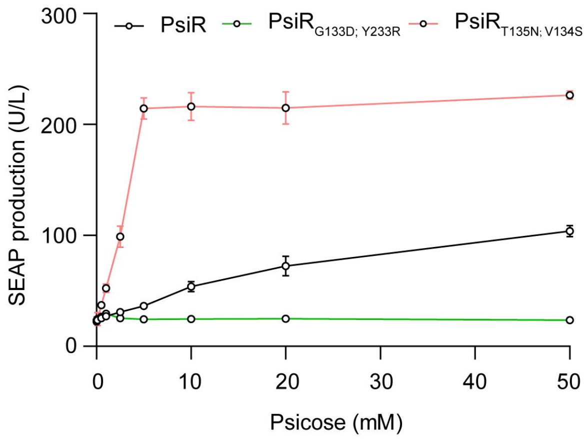

Supplementary Figure 3 Dose-dependent psicose-induced SEAP expression using PsiR$_{WT}$, PsiR$_{G133D;\ Y233R}$, and PsiR$_{T135N;V134S}$. HEK-293T cells were co-transfected with pQL164 and either pQL172, or pQL173, or pQL656 (P$_{hEF1\alpha}$-KRAB-PsiR$_{G133D;\ Y233R}$-pA). SEAP production was quantified 48 hours after cultivation with different concentrations of psicose. Data are presented as means ± SD; n = 3 independent experiments. Detailed descriptions of the genetic constructs and transfection mixtures are provided in **Supplementary Tables 1 and 3**.

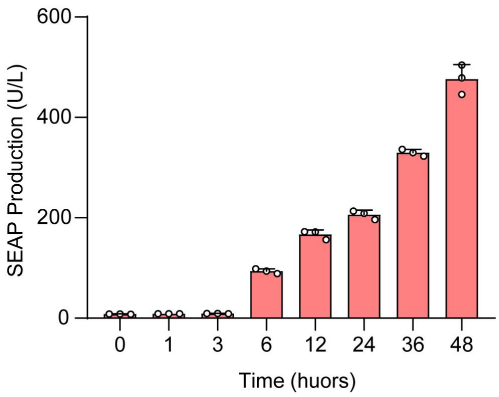

Supplementary Figure 4 Time dependent SEAP expression kinetics of the PURE system. SEAP levels of stable cell line $HEK_{PURE\text{-}SEAP}$, exposed to 5 mM psicose for different time periods (0 to 48 hours), were profiled at 48 hours. All data are presented as means ± SD; n = 3 independent experiments.

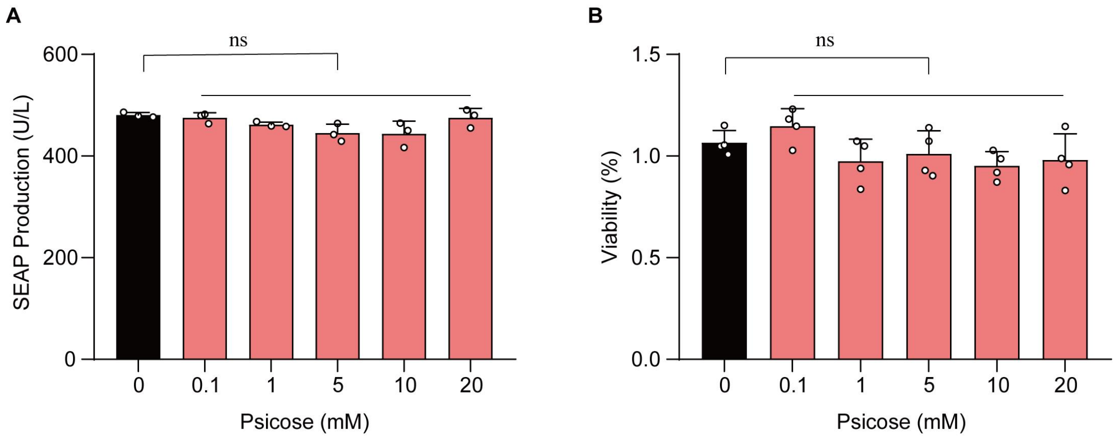

Supplementary Figure 5 Psicose toxicity on HEK-293T cells. a, Gene expression-based metabolic integrity assay of HEK-293T cells after exposure to various concentrations of psicose. HEK-293T cells (5 × 10^4) were transfected with pSEAP2-Control (P_{hCMV}-SEAP-pA, 100 ng), and cultivated with different concentrations of psicose. SEAP expression in the culture supernatants was profiled after 48 hours incubation. Data are expressed as mean ± SD, n = 3 independent experiments. **b,** Cell viability of HEK-293T cells exposed to various concentrations of psicose. HEK-293T cells (1×10^4) were cultivated in medium containing various concentrations of psicose, and cell viability was measured by MTT assay at 48 hours. Data are expressed as mean ± SD, n = 4 independent experiments. *P* values in a were calculated by one-way ANOVA with multiple comparisons. ns, not significant. Detailed descriptions of the genetic constructs and transfection mixtures are provided in **Supplementary Tables 1 and 3**.

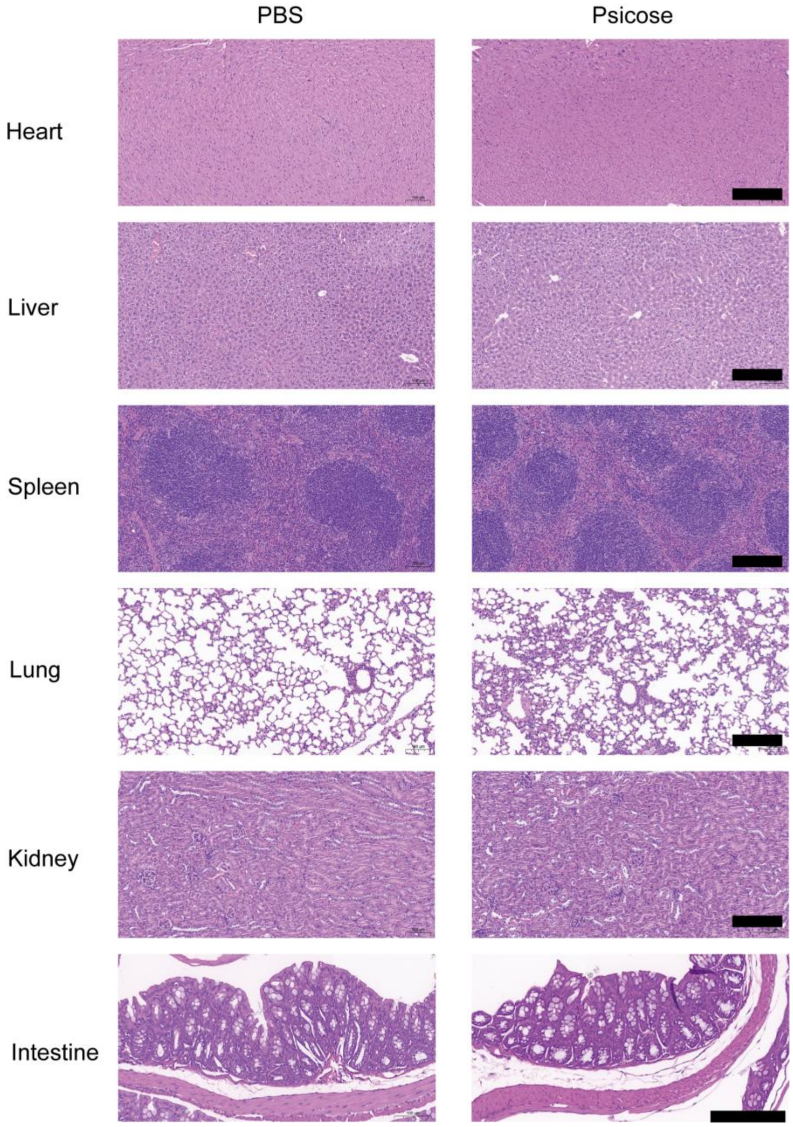

Supplementary Figure 6 Representative H&E staining of major organs and intestinal tissues of C57BL/6 mice after oral administration of psicose. Healthy wild-type C57BL/6 mice were randomly assigned to two groups and orally administered either psicose (1 g/kg) or an equal volume of PBS daily for two weeks. Major organs (heart, liver, spleen, lung, kidney) and intestinal tissues were collected and stained by H&E. Scale bar, 200 μm.

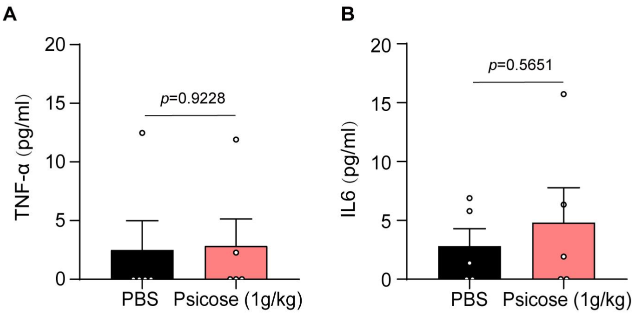

Supplementary Figure 7 Inflammatory cytokine levels of C57BL/6 mice after oral administration of psicose. Healthy wild-type C57BL/6 mice were randomly assigned to two groups and orally administered either psicose (1 g/kg) or an equal volume of PBS daily for two weeks. Blood was collected, and serum inflammatory cytokine TNF-α (**A**) and IL-6 (**B**) levels were quantified using ELISA. Data are presented as the means ± SEM; $n = 5$ mice. P values were calculated by a two-tailed unpaired t-test.

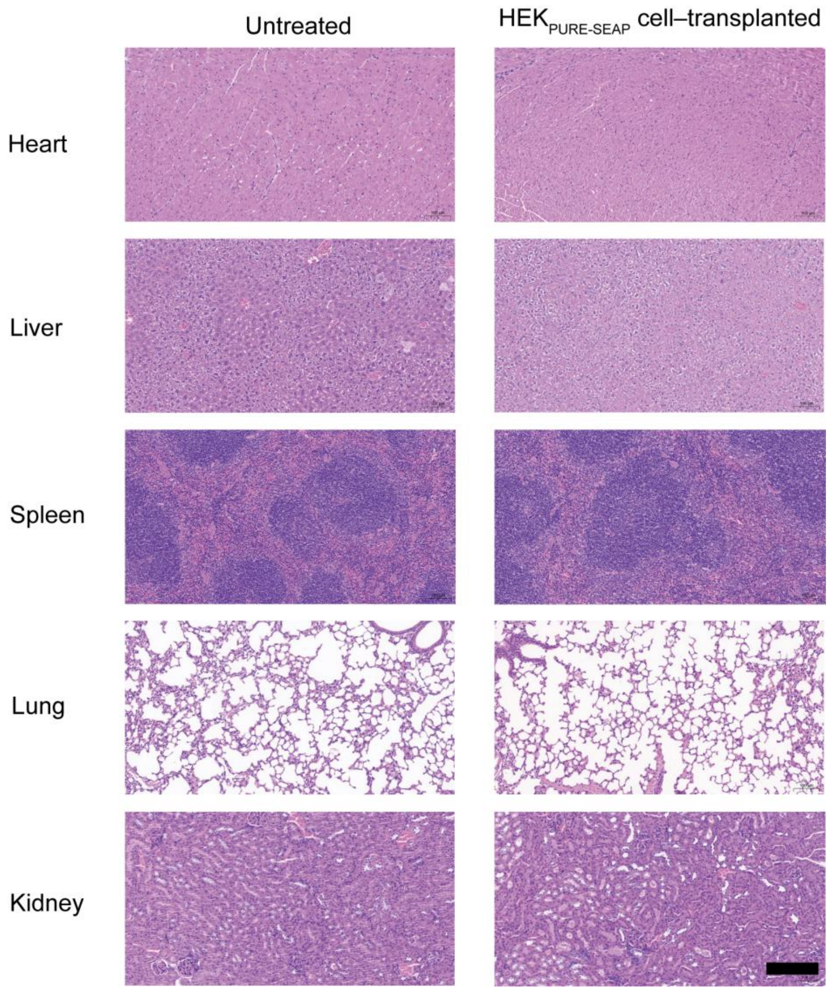

Supplementary Figure 8 Representative H&E staining of major organs from C57BL/6 mice after transplantation of HEK$_{PURE\text{-}SEAP}$ cells. The microencapsulated HEK$_{PURE\text{-}SEAP}$ cells were implanted into the peritoneal cavity for two weeks. Major organs (heart, liver, spleen, lung, kidney) were collected and stained by H&E. Scale bar, 200 μm.

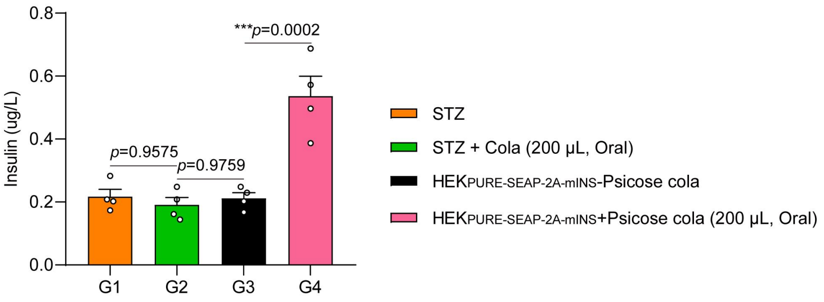

Supplementary Figure 9 Insulin production of PURE designer cells in T1D mice by drinking psicose cola. Streptozotocin (STZ)-induced T1D mice were randomly allocated into four experimental groups: G1, STZ-induced T1D control mice (STZ); G2, STZ-induced T1D mice implanted with microencapsulated $\text{HEK}_{\text{PURE-SEAP-2A-mINS}}$ cells and exposed to cola ($\text{HEK}_{\text{PURE-SEAP-2A-mINS}}$ + psicose cola); G3, STZ-induced T1D mice implanted with microencapsulated $\text{HEK}_{\text{PURE-SEAP-2A-mINS}}$ cells without cola exposure ($\text{HEK}_{\text{PURE-SEAP-2A-mINS}}$ – psicose cola); and G4, STZ-induced T1D mice implanted with microencapsulated $\text{HEK}_{\text{PURE-SEAP-2A-mINS}}$ cells and exposed to psicose cola ($\text{HEK}_{\text{PURE-SEAP-2A-mINS}}$ + psicose cola). Mice in groups G2 and G4 received daily oral administration of 200 μL sugar-free cola or psicose cola (corresponding to 1 g psicose per kg body weight). Serum insulin levels were profiled after 24 hours. Data are presented as the means ± SEM; $n = 4$ mice. P values were calculated by one-way ANOVA with multiple comparisons. ***$P < 0.001$.

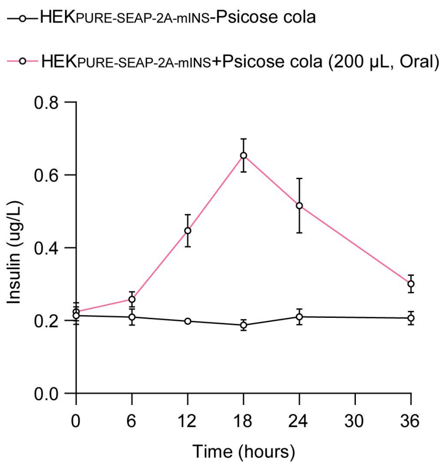

Supplementary Figure 10 The kinetic parameters of insulin expression activated by the PURE system upon psicose induction. STZ-induced T1D mice were implanted with microencapsulated $HEK_{PURE\text{-}SEAP\text{-}2A\text{-}mINS}$ cells and exposed or unexposed to psicose cola (corresponding to 1 g psicose per kg body weight, 200 µL). Serum insulin levels were measured at different time points (0-36 hours). Data are presented as the means ± SEM; $n = 4$ mice.

Table S1. Plasmids designed and used in this study

Plasmid	Description and cloning strategy	Reference
pcDNA3.1(+)	Constitutive mammalian P_{hCMV}-driven expression vector (P_{hCMV}-MCS-pA)	Invitrogen' CA
SB100X	Constitutive P_{hCMV}-driven transposase SB100X expression vector (P_{hCMV}-SB100X-pA)	Addgene (no. 13367)
pSEAP2-Control	Constitutive SEAP fusion protein expression vector (P_{hCMV}-SEAP-pA)	This study
pQL162	Psicose induced SEAP expression vector (P_{PsiR1}-SEAP-pA; P_{PsiR1}, $P_{hCMV\text{-}(PsiO)1}$)	This study
pQL163	Psicose induced SEAP expression vector (P_{PsiR2}-SEAP-pA; P_{PsiR2}, $P_{hCMV\text{-}(PsiO)2}$)	This study
pQL164	Psicose induced SEAP expression vector (P_{PsiR3}-SEAP-pA; P_{PsiR3}, $P_{hCMV\text{-}(PsiO)3}$)	This study
pQL165	Psicose induced SEAP expression vector (P_{PsiR4}-SEAP-pA; P_{PsiR4}, $P_{hCMV\text{-}(PsiO)4}$)	This study
pQL166	Psicose induced SEAP expression vector (P_{PsiR5}-SEAP-pA; P_{PsiR5}, $P_{hCMV\text{-}(PsiO)1}$)	This study
pQL172	Constitutive KRAB-PsiR fusion protein expression vector ($P_{hEF1\alpha}$-KRAB-PsiR-pA)	This study
pQL173	Constitutive KRAB-$PsiR_{T135N;V134S}$ fusion protein expression vector ($P_{hEF1\alpha}$-KRAB-$PsiR_{T135N;V134S}$-pA)	This study
pQL174	Constitutive KRAB-$PsiR_{T135N;V134S}$ fusion protein expression vector ($P_{hEF1\alpha}$-KRAB-$PsiR_{T135N}$-pA)	This study
pQL450	Sleeping Beauty transposase expression vector encoding constitutive KRAB-$PsiR_{T135N;V134S}$ fusion protein utilizing a PuroR resistance cassette (ITR-$P_{hEF1\alpha}$-KRAB-$PsiR_{T135N;V134S}$-pA :: P_{mPGK}-PuroR-pA-ITR)	This study
pQL451	Sleeping Beauty transposase expression vector encoding psicose induced SEAP and mINS utilizing a ZeoR resistance cassette (ITR-P_{PsiR3}-SEAP-P2A-mINS-pA::P_{mPGK}-ZeoR-P2A-EG	This study

	FP-pA-ITR)	
pQL452	Sleeping Beauty transposase expression vector encoding psicose induced SEAP and mTSLP utilizing a ZeoR resistance cassette (ITR-P_{PsiR3}-mTSLP-pA::P_{mPGK}-ZeoR-P2A-EGFP-pA-ITR)	This study
pQL656	Constitutive KRAB-$PsiR_{G133D;Y233R}$ fusion protein expression vector ($P_{hEF1\alpha}$-KRAB-$PsiR_{G133D;Y233R}$-pA)	This study

Abbreviations: **BAT,** brown adipose tissue; **DMSO,** dimethylsulfoxide; **eWAT,** epididymal white adipose tissue; **FDA**, Food and Drug Administration; **GOI,** gene of interest; **GRAS,** Generally Recognized As Safe; **H&E,** Histological examination; **HFD,** high-fat diet; **KRAB,** the human Krüppel-associated box domain, **i.p.,** intraperitoneal; **IPGTT,** intraperitoneal glucose tolerance test; **ITT,** insulin tolerance test; **iWAT,** subcutaneous inguinal white adipose tissue; **KRAB,** human Krueppel-associated box; **mINS,** mouse insulin; **mTSLP,** mouse thymic stromal lymphopoietin; **PsiR,** a LacI-family transcriptional regulator with high affinity for psicose; **PsiO,** PsiR binding site; **PTH,** parathyroid hormone; **PURE**, psicose-inducible transgene expression; **SEAP,** secreted embryonic alkaline phosphatase; **SB,** Sleeping Beauty transposase; **STZ,** Streptozotocin; **T1D,** type 1 diabetic model; **TGs,** triglycerides; **WT,** wild-type.

Table S2. Expression vectors and transfection mixtures used in Main Figures.

Plasmid (ng)	Fig. 1b	Fig. 1d	Fig. 1e	Fig. 1f PsiR	Fig. 1f $PsiR_{T135N}$	Fig. 1f $PsiR_{T135N;V134S}$
pQL164	100	100	100	100	100	100
pQL172	100	0	0	100	0	0
pQL172 $PsiR_{T135X}$	0	100	0	0	0	0
$PsiR_{T135N}$	0	0	0	0	100	0
$PsiR_{T135N;V134X}$	0	0	100	0	0	0
$PsiR_{T135N;V134S}$	0	0	150	0	0	100

Total mount	200	200	200	200	200	200

Plasmid (ng)	Fig. 2a	Fig. 2b
pQL164	100	100
pQL173	100	100
Total amount	200	200

Table S3. Expression vectors and transfection mixtures used in **Supplementary Figs.**

Plasmid (ng)	Fig. S1a	Fig. S1b	Fig. S1c	Fig. S1d
pQL172	100	100	100	100
pQL162	100	0	0	0
pQL163	0	100	0	0
pQL165	0	0	100	0
pQL166	0	0	0	100
Total mount	200	200	200	200

Plasmid (ng)	Fig. S2a	Fig. S2b	Fig. S2c
pQL164	100	100	100
$PsiR_{T135N;V134S;P137X}$	100	0	0
$PsiR_{T135N;V134S;L156X}$	0	100	0
$PsiR_{T135N;V134S;P157X}$	0	0	100
Total mount	200	200	200

Plasmid (ng)	Fig. S3	Fig. S3	Fig. S3
pQL164	100	100	100
pQL172	100	0	0
pQL173	0	100	0
pQL656	0	0	100
Total mount	200	200	200

Plasmid (ng)	Fig. S5a
pSEAP2-Control	100

Total amount	100

Table S4. DNA sequence information of the PURE system

**pQL173: $P_{hEF1\alpha}$-KRAB-$PsiR_{T135N;V134S}$-pA.** The gray highlight indicates the positions of the T135N and V134S mutations.
GGGAAAGTGATGTCGTGTACTGGCTCCGCCTTTTTCCCGAGGGTGGGGGAGAACCG<br>TATATAAGTGCAGTAGTCGCCGTGAACGTTCTTTTTCGCAACGGGTTTGCCGCCAGA<br>ACACAGGTAAGTGCCGTGTGTGGTTCCCGCGGGCCTGGCCTCTTTACGGGTTATGGC<br>CCTTGCGTGCCTTGAATTACTTCCACCTGGCTGCAGTACGTGATTCTTGATCCCGAG<br>CTTCGGGTTGGAAGTGGGTGGGAGAGTTCGAGGCCTTGCGCTTAAGGAGCCCCTTC<br>GCCTCGTGCTTGAGTTGAGGCCTGGCCTGGGCGCTGGGGCCGCCGCGTGCGAATCT<br>GGTGGCACCTTCGCGCCTGTCTCGCTGCTTTCGATAAGTCTCTAGCCATTTAAAATTT<br>TTGATGACCTGCTGCGACGCTTTTTTTCTGGCAAGATAGTCTTGTAAATGCGGGCCA<br>AGATCTGCACACTGGTATTTCGGTTTTTGGGGCCGCGGGCGGCGACGGGGCCCGTG<br>CGTCCCAGCGCACATGTTCGGCGAGGCGGGGCCTGCGAGCGCGGCCACCGAGAATC<br>GGACGGGGGTAGTCTCAAGCTGGCCGGCCTGCTCTGGTGCCTGGCCTCGCGCCGCC<br>GTGTATCGCCCCGCCCTGGGCGGCAAGGCTGGCCCGGTCGGCACCAGTTGCGTGAG<br>CGGAAAGATGGCCGCTTCCCGGCCCTGCTGCAGGGAGCTCAAAATGGAGGACGCGG<br>CGCTCGGGAGAGCGGGCGGGTGAGTCACCCACACAAAGGAAAAGGGCCTTTCCGTC<br>CTCAGCCGTCGCTTCATGTGACTCCACGGAGTACCGGGCGCCGTCCAGGCACCTCGA<br>TTAGTTCTCGAGCTTTTGGAGTACGTCGTCTTTAGGTTGGGGGGAGGGGTTTTATGC<br>GATGGAGTTTCCCCACACTGAGTGGGTGGAGACTGAAGTTAGGCCAGCTTGGCACTT<br>GATGTAATTCTCCTTGGAATTTGCCCTTTTTGAGTTTGGATCTTGGTTCATTCTCAAG<br>CCTCAGACAGTGGTTCAAAGTTTTTTTCTTCCATTTCAGGTGTCGTGAGGAATTAGCT<br>TGGTAGCTAGCGCCACCATGGCGCCAGATCCAAAAAAGAAGAGAAAGGTAGATCCA<br>AAAAAGAAGAGAAAGGTAGATCCAAAAAAGAAGAGAAAGGTAATGGATGCTAAGTC<br>ACTAACTGCCTGGTCCCGGACACTGGTGACCTTCAAGGATGTATTTGTGGACTTCAC<br>CAGGGAGGAGTGGAAGCTGCTGGACACTGCTCAGCAGATCGTGTACAGAAATGTGA<br>TGCTGGAGAACTATAAGAACCTGGTTTCCTTGGGTTATCAGCTTACTAAGCCAGATG<br>TGATCCTCCGGTTGGAGAAGGGAGAAGAGCCCTGGCTGGTGGAGAGAGAAATTCAC<br>CAAGAGACCCATCCTGATTCAGAGACTGCATTTGAAATCAAATCATCAGTTTCCAGC<br>AGGAGCATTTTTAAAGATAAGCAATCCTGTGACATTAAAATGGAAGGAATGGCAAGG<br>AATGATCTCTGGGCCAGCGGAAGTGGCGGAGGAGGCGACGTCATGACAGGCATCAG<br>CAGCAAGAAGGCCACCATCTACGACCTGAGCATCCTGTCTGGCGCCTCTGCCTCTAC<br>AGTGTCCGCTGTGCTGAACGGCTCTTGGCGGAAGCGGAGAATCAGCGAGGAAACCG<br>CCGACAAGATCCTGAGCCTGGCTAAGGCCCAGCGGTACACCACAAATCTGCAGGCC<br>AGAGGCCTGCGGTCTAGCAAGTCTGGACTTGTGGGACTGCTGGTGCCCGTGTACGA<br>CAACAGATTCTTCAGCAGCATGGCCCAGACCTTTGAAGGCCAGGCCAGAAAGCGAG<br>GACTGAGCCCTATGGTGGTGTCCGGCAGAAGAGATCCCGAAGAGGAACGCAGAACC<br>GTGGAAACCCTGATCGCCTACTCCATCGACGCCCTGTTTATCGCCGGCAGCAATGAT<br>CCTGATGGCGTGCACCAAGTGTGCGCCAGAGCTGCTCTGCCTCACGTGAACATCGAT<br>CTGCCTGGCAAGTTCGCCAGCAGCGTGATCAGCAACAATAGACACGGCGCCGAGAT<br>CCTGACCGCCGCCATTCTTGCTCATGCCGCCAAAGGCGGATCTCTGGGCCCTGATGA<br>TGTGATCCTGTTCGGCGGCCACGATGACCACGCCAGCAGAGAAAGAATCGACGGCT<br>TTCACGCCGCCAAGGCCGATTACTTTGGAGTGGAAGGCGGCGACGACATCGAGATC<br>ACAGGATACAGCCCTCACATGACCGAGATGGCCTTCGAGCGGTTCTTCGGCAGAAG<br>GGGCAGACTGCCCAGATGCTTCTTCGTGAACAGCAGCATCAACTTCGAGGGCCTGCT<br>GCGGTTCATGGGCAGACATGATGGCGAGGCCTTCGGCGATATCGTCGTGGGCTGCT<br>TCGACTACGATCCCTTCGCCAGCTTCCTGCCTTTTCCTGTGTACATGATCAAGCCCGA<br>TATCGCTCAGATGCTGGAAAAGGGCTTCGAGCTGCTGGAAGAGAACCGGACCGAGC<br>CTGAAGTGACCATCATCGAGCCTCAGCTGATCCCTCCTAGAACAGCCCTGGAAGGCC<br>CTCTGGACGACATCTGGGATCCTGTGGCTCTGCGGAGAATGGCCAAGTGA
**pQL164: P_{PsiR3}-SEAP-pA; P_{PsiR3}, $P_{hCMV-(PsiO)3}$.** The grey highlight indicates three copies of the PsiO sequence.
GTTGACATTGATTATTGACTAGTTATTAATAGTAATCAATTACGGGGTCATTAGTTCA<br>TAGCCCATATATGGAGTTCCGCGTTACATAACTTACGGTAAATGGCCCGCCTGGCTG<br>ACCGCCCAACGACCCCCGCCCATTGACGTCAATAATGACGTATGTTCCCATAGTAAC

GCCAATAGGGACTTTCCATTGACGTCAATGGGTGGAGTATTTACGGTAAACTGCCCA
CTTGGCAGTACATCAAGTGTATCATATGCCAAGTACGCCCCCTATTGACGTCAATGA
CGGTAAATGGCCCGCCTGGCATTATGCCCAGTACATGACCTTATGGGACTTTCCTAC
TTGGCAGTACATCTACGTATTAGTCATCGCTATTACCATGGTGATGCGGTTTTGGCA
GTACATCAATGGGCGTGGATAGCGGTTTGACTCACGGGGATTTCCAAGTCTCCACCC
CATTGACGTCAATGGGAGTTTGTTTTGGCACCAAAATCAACGGGACTTTCCAAAATG
TCGTAACAACTCCGCCCCATTGACGCAAATGGGCGGTAGGCGTGTACGGTGGGAGG
TCTATATAAGCAGAGCTCTCTGGCTAACTAGAGAACCCACTGCTTACTGGCTTATCG
AAATTAATACGACTCACTATAGGGAGACCCAAGCTGGCTAGCGTTTAAACTTAAGCT
TGGTACCATTGCACAATCGATGGTGCAAATTGCACAATCGATGGTGCAAATTGCACA
ATCGATGGTGCAAGGCCACCATGCTGCTGCTGCTGCTGCTGCTGGGCCTGAGGCTAC
AGCTCTCCCTGGGCATCATCCCAGTTGAGGAGGAGAACCCGGACTTCTGGAACCGC
GAGGCAGCCGAGGCCCTGGGTGCCGCCAAGAAGCTGCAGCCTGCACAGACAGCCGC
CAAGAACCTCATCATCTTCCTGGGCGATGGGATGGGGGTGTCTACGGTGACAGCTGC
CAGGATCCTAAAAGGGCAGAAGAAGGACAAACTGGGGCCTGAGATACCCCTGGCCA
TGGACCGCTTCCCATATGTGGCTCTGTCCAAGACATACAATGTAGACAAACATGTGC
CAGACAGTGGAGCCACAGCCACGGCCTACCTGTGCGGGGTCAAGGGCAACTTCCAG
ACCATTGGCTTGAGTGCAGCCGCCCGCTTTAACCAGTGCAACACGACACGCGGCAAC
GAGGTCATCTCCGTGATGAATCGGGCCAAGAAAGCAGGGAAGTCAGTGGGAGTGGT
AACCACCACACGAGTGCAGCACGCCTCGCCAGCCGGCACCTACGCCCACACGGTGA
ACCGCAACTGGTACTCGGACGCCGACGTGCCTGCCTCGGCCCGCCAGGAGGGGTGC
CAGGACATCGCTACGCAGCTCATCTCCAACATGGACATTGACGTGATCCTAGGTGGA
GGCCGAAAGTACATGTTTCGCATGGGAACCCCAGACCCTGAGTACCCAGATGACTAC
AGCCAAGGTGGGACCAGGCTGGACGGGAAGAATCTGGTGCAGGAATGGCTGGCGAA
GCGCCAGGGTGCCCGGTATGTGTGGAACCGCACTGAGCTCATGCAGGCTTCCCTGG
ACCCGTCTGTGACCCATCTCATGGGTCTCTTTGAGCCTGGAGACATGAAATACGAGA
TCCACCGAGACTCCACACTGGACCCCTCCCTGATGGAGATGACAGAGGCTGCCCTGC
GCCTGCTGAGCAGGAACCCCCGCGGCTTCTTCCTCTTCGTGGAGGGTGGTCGCATCG
ACCATGGTCATCATGAAAGCAGGGCTTACCGGGCACTGACTGAGACGATCATGTTCG
ACGACGCCATTGAGAGGGCGGGCCAGCTCACCAGCGAGGAGGACACGCTGAGCCTC
GTCACTGCCGACCACTCCCACGTCTTCTCCTTCGGAGGCTACCCCCTGCGAGGGAGC
TCCATCTTCGGGCTGGCCCCTGGCAAGGCCCGGGACAGGAAGGCCTACACGGTCCT
CCTATACGGAAACGGTCCAGGCTATGTGCTCAAGGACGGCGCCCGGCCGGATGTTA
CCGAGAGCGAGAGCGGGAGCCCCGAGTATCGGCAGCAGTCAGCAGTGCCCCTGGAC
GAAGAGACCCACGCAGGCGAGGACGTGGCGGTGTTCGCGCGCGGCCCGCAGGCGC
ACCTGGTTCACGGCGTGCAGGAGCAGACCTTCATAGCGCACGTCATGGCCTTCGCCG
CCTGCCTGGAGCCCTACACCGCCTGCGACCTGGCGCCCCCCGCCGGCACCACCGAC
GCCGCGCACCCGGGTTACTCTAGAGTCGGGGCGGCCGGCCGCTTCGAGCAGACATG
A

pQL450: ITR-$P_{hEF1\alpha}$-KRAB-$PsiR_{T135N;V134S}$-pA::P_{mPGK}-PuroR-pA-ITR

GTAAGTGCCGTGTGTGGTTCCCGCGGGCCTGGCCTCTTTACGGGTTATGGCCCTTGC
GTGCCTTGAATTACTTCCACCTGGCTGCAGTACGTGATTCTTGATCCCGAGCTTCGG
GTTGGAAGTGGGTGGGAGAGTTCGAGGCCTTGCGCTTAAGGAGCCCCTTCGCCTCG
TGCTTGAGTTGAGGCCTGGCCTGGGCGCTGGGGCCGCCGCGTGCGAATCTGGTGGC
ACCTTCGCGCCTGTCTCGCTGCTTTCGATAAGTCTCTAGCCATTTAAAATTTTTGATG
ACCTGCTGCGACGCTTTTTTTCTGGCAAGATAGTCTTGTAAATGCGGGCCAAGATCT
GCACACTGGTATTTCGGTTTTTGGGGCCGCGGGCGGCGACGGGGCCCGTGCGTCCC
AGCGCACATGTTCGGCGAGGCGGGGCCTGCGAGCGCGGCCACCGAGAATCGGACGG
GGGTAGTCTCAAGCTGGCCGGCCTGCTCTGGTGCCTGGCCTCGCGCCGCCGTGTAT
CGCCCCGCCCTGGGCGGCAAGGCTGGCCCGGTCGGCACCAGTTGCGTGAGCGGAAA
GATGGCCGCTTCCCGGCCCTGCTGCAGGGAGCTCAAAATGGAGGACGCGGCGCTCG
GGAGAGCGGGCGGGTGAGTCACCCACACAAAGGAAAAGGGCCTTTCCGTCCTCAGC
CGTCGCTTCATGTGACTCCACGGAGTACCGGGCGCCGTCCAGGCACCTCGATTAGTT
CTCGAGCTTTTGGAGTACGTCGTCTTTAGGTTGGGGGGAGGGGTTTTATGCGATGGA
GTTTCCCCACACTGAGTGGGTGGAGACTGAAGTTAGGCCAGCTTGGCACTTGATGTA
ATTCTCCTTGGAATTTGCCCTTTTTGAGTTTGGATCTTGGTTCATTCTCAAGCCTCAG
ACAGTGGTTCAAAGTTTTTTTCTTCCATTTCAGGTGTCGTGAGGAATTAGCTTGGTAG
CTAGCGCCACCATGGCGCCAGATCCAAAAAAGAAGAGAAAGGTAGATCCAAAAAAG
AAGAGAAAGGTAGATCCAAAAAAGAAGAGAAAGGTAATGGATGCTAAGTCACTAACT
GCCTGGTCCCGGACACTGGTGACCTTCAAGGATGTATTTGTGGACTTCACCAGGGAG

GAGTGGAAGCTGCTGGACACTGCTCAGCAGATCGTGTACAGAAATGTGATGCTGGA
GAACTATAAGAACCTGGTTTCCTTGGGTTATCAGCTTACTAAGCCAGATGTGATCCT
CCGGTTGGAGAAGGGAGAAGAGCCCTGGCTGGTGGAGAGAGAAATTCACCAAGAGA
CCCATCCTGATTCAGAGACTGCATTTGAAATCAAATCATCAGTTTCCAGCAGGAGCA
TTTTTAAAGATAAGCAATCCTGTGACATTAAAATGGAAGGAATGGCAAGGAATGATC
TCTGGGCCAGCGGAAGTGGCGGAGGAGGCGACGTCATGACAGGCATCAGCAGCAAG
AAGGCCACCATCTACGACCTGAGCATCCTGTCTGGCGCCTCTGCCTCTACAGTGTCC
GCTGTGCTGAACGGCTCTTGGCGGAAGCGGAGAATCAGCGAGGAAACCGCCGACAA
GATCCTGAGCCTGGCTAAGGCCCAGCGGTACACCACAAATCTGCAGGCCAGAGGCC
TGCGGTCTAGCAAGTCTGGACTTGTGGGACTGCTGGTGCCCGTGTACGACAACAGAT
TCTTCAGCAGCATGGCCCAGACCTTTGAAGGCCAGGCCAGAAAGCGAGGACTGAGC
CCTATGGTGGTGTCCGGCAGAAGAGATCCCGAAGAGGAACGCAGAACCGTGGAAAC
CCTGATCGCCTACTCCATCGACGCCCTGTTTATCGCCGGCAGCAATGATCCTGATGG
CGTGCACCAAGTGTGCGCCAGAGCTGCTCTGCCTCACGTGAACATCGATCTGCCTGG
CAAGTTCGCCAGCAGCGTGATCAGCAACAATAGACACGGCGCCGAGATCCTGACCG
CCGCCATTCTTGCTCATGCCGCCAAAGGCGGATCTCTGGGCCCTGATGATGTGATCC
TGTTCGGCGGCCACGATGACCACGCCAGCAGAGAAAGAATCGACGGCTTTCACGCC
GCCAAGGCCGATTACTTTGGAGTGGAAGGCGGCGACGACATCGAGATCACAGGATA
CAGCCCTCACATGACCGAGATGGCCTTCGAGCGGTTCTTCGGCAGAAGGGGCAGAC
TGCCCAGATGCTTCTTCGTGAACAGCAGCATCAACTTCGAGGGCCTGCTGCGGTTCA
TGGGCAGACATGATGGCGAGGCCTTCGGCGATATCGTCGTGGGCTGCTTCGACTAC
GATCCCTTCGCCAGCTTCCTGCCTTTTCCTGTGTACATGATCAAGCCCGATATCGCTC
AGATGCTGGAAAAGGGCTTCGAGCTGCTGGAAGAGAACCGGACCGAGCCTGAAGTG
ACCATCATCGAGCCTCAGCTGATCCCTCCTAGAACAGCCCTGGAAGGCCCTCTGGAC
GACATCTGGGATCCTGTGGCTCTGCGGAGAATGGCCAAGTGAGGTACCTCACTGACC
TTCAAGAAACGTAATTAAACGCGTCAGACATGATAAGATACATTGATGAGTTTGGAC
AAACCACAACTAGAATGCAGTGAAAAAAATGCTTTATTTGTGAAATTTGTGATGCTAT
TGCTTTATTTGTAACCATTATAAGCTGCAATAAACAAGTTAACAACAACAATTGCATT
CATTTTATGTTTCAGGTTCAGGGGGAGGTGTGGGAGGTTTTTTAAAGCAAGTAAAAC
CTCTACAAATGTGGTATGGCTGATTATGATCCTGCCCGGGTAGGGGAGGCGCTTTTC
CCAAGGCAGTCTGGAGCATGCGCTTTAGCAGCCCCGCTGGGCACTTGGCGCTACAC
AAGTGGCCTCTGGCCTCGCACACATTCCACATCCACCGGTAGGCGCCAACCGGCTCC
GTTCTTTGGTGGCCCCTTCGCGCCACCTTCTACTCCTCCCCTAGTCAGGAAGTTCCC
CCCCGCCCCGCAGCTCGCGTCGTGCAGGACGTGACAAATGGAAGTAGCACGTCTCA
CTAGTCTCGTGCAGATGGACAGCACCGCTGAGCAATGGAAGCGGGTAGGCCTTTGG
GGCAGCGGCCAATAGCAGCTTTGCTCCTTCGCTTTCTGGGCTCAGAGGCTGGGAAG
GGGTGGGTCCGGGGGCGGGCTCAGGGGCGGGCTCAGGGGCGGGGCGGGCGCCCGA
AGGTCCTCCGGAGGCCCGGCATTCTGCACGCTTCAAAAGCGCACGTCTGCCGCGCT
GTTCTCCTCTTCCTCATCTCCGGGCCTTTCGACCTGCAGCCCAAGCTTACCATGACC
GAGTACAAGCCCACGGTGCGCCTCGCCACCCGCGACGACGTCCCCAGGGCCGTACG
CACCCTCGCCGCCGCGTTCGCCGACTACCCCGCCACGCGCCACACCGTCGATCCGG
ACCGCCACATCGAGCGGGTCACCGAGCTGCAAGAACTCTTCCTCACGCGCGTCGGG
CTCGACATCGGCAAGGTGTGGGTCGCGGACGACGGCGCCGCGGTGGCGGTCTGGAC
CACGCCGGAGAGCGTCGAAGCGGGGGCGGTGTTCGCCGAGATCGGCCCGCGCATGG
CCGAGTTGAGCGGTTCCCGGCTGGCCGCGCAGCAACAGATGGAAGGCCTCCTGGCG
CCGCACCGGCCCAAGGAGCCCGCGTGGTTCCTGGCCACCGTCGGCGTCTCGCCCGA
CCACCAGGGCAAGGGTCTGGGCAGCGCCGTCGTGCTCCCCGGAGTGGAGGCGGCCG
AGCGCGCCGGGGTGCCCGCCTTCCTGGAGACCTCCGCGCCCCGCAACCTCCCCTTCT
ACGAGCGGCTCGGCTTCACCGTCACCGCCGACGTCGAGGTGCCCGAAGGACCGCGC
ACCTGGTGCATGACCCGCAAGCCCGGTGCCTGA

pQL451: ITR-P_{PsiR3}-SEAP-P2A-mINS-pA::P_{mPGK}-ZeoR-P2A-EGFP-pA-ITR.
The grey highlight indicates three copies of the PsiO sequence.

GACATTGATTATTGACTAGTTATTAATAGTAATCAATTACGGGGTCATTAGTTCATAG
CCCATATATGGAGTTCCGCGTTACATAACTTACGGTAAATGGCCCGCCTGGCTGACC
GCCCAACGACCCCCGCCCATTGACGTCAATAATGACGTATGTTCCCATAGTAACGCC
AATAGGGACTTTCCATTGACGTCAATGGGTGGAGTATTTACGGTAAACTGCCCACTT
GGCAGTACATCAAGTGTATCATATGCCAAGTACGCCCCCTATTGACGTCAATGACGG
TAAATGGCCCGCCTGGCATTATGCCCAGTACATGACCTTATGGGACTTTCCTACTTG
GCAGTACATCTACGTATTAGTCATCGCTATTACCATGGTGATGCGGTTTTGGCAGTA
CATCAATGGGCGTGGATAGCGGTTTGACTCACGGGGATTTCCAAGTCTCCACCCCAT

TGACGTCAATGGGAGTTTGTTTTGGCACCAAAATCAACGGGACTTTCCAAAATGTCG
TAACAACTCCGCCCCATTGACGCAAATGGGCGGTAGGCGTGTACGGTGGGAGGTCT
ATATAAGCAGAGCTCTCTGGCTAACTAGAGAACCCACTGCTTACTGGCTTATCGAAA
TTAATACGACTCACTATAGGGAGACCCAAGCTGGCTAGCGTTTAAACTTAAGCTTGG
TACCATTGCACAATCGATGGTGCAAATTGCACAATCGATGGTGCAAATTGCACAATC
GATGGTGCAAGGCCACCATGCTGCTGCTGCTGCTGCTGCTGGGCCTGAGGCTACAG
CTCTCCCTGGGCATCATCCCAGTTGAGGAGGAGAACCCGGACTTCTGGAACCGCGA
GGCAGCCGAGGCCCTGGGTGCCGCCAAGAAGCTGCAGCCTGCACAGACAGCCGCCA
AGAACCTCATCATCTTCCTGGGCGATGGGATGGGGGTGTCTACGGTGACAGCTGCCA
GGATCCTAAAAGGGCAGAAGAAGGACAAACTGGGGCCTGAGATACCCCTGGCCATG
GACCGCTTCCCATATGTGGCTCTGTCCAAGACATACAATGTAGACAAACATGTGCCA
GACAGTGGAGCCACAGCCACGGCCTACCTGTGCGGGGTCAAGGGCAACTTCCAGAC
CATTGGCTTGAGTGCAGCCGCCCGCTTTAACCAGTGCAACACGACACGCGGCAACGA
GGTCATCTCCGTGATGAATCGGGCCAAGAAAGCAGGGAAGTCAGTGGGAGTGGTAA
CCACCACACGAGTGCAGCACGCCTCGCCAGCCGGCACCTACGCCCACACGGTGAAC
CGCAACTGGTACTCGGACGCCGACGTGCCTGCCTCGGCCCGCCAGGAGGGGTGCCA
GGACATCGCTACGCAGCTCATCTCCAACATGGACATTGACGTGATCCTAGGTGGAGG
CCGAAAGTACATGTTTCGCATGGGAACCCCAGACCCTGAGTACCCAGATGACTACAG
CCAAGGTGGGACCAGGCTGGACGGGAAGAATCTGGTGCAGGAATGGCTGGCGAAGC
GCCAGGGTGCCCGGTATGTGTGGAACCGCACTGAGCTCATGCAGGCTTCCCTGGAC
CCGTCTGTGACCCATCTCATGGGTCTCTTTGAGCCTGGAGACATGAAATACGAGATC
CACCGAGACTCCACACTGGACCCCTCCCTGATGGAGATGACAGAGGCTGCCCTGCG
CCTGCTGAGCAGGAACCCCCGCGGCTTCTTCCTCTTCGTGGAGGGTGGTCGCATCGA
CCATGGTCATCATGAAAGCAGGGCTTACCGGGCACTGACTGAGACGATCATGTTCGA
CGACGCCATTGAGAGGGCGGGCCAGCTCACCAGCGAGGAGGACACGCTGAGCCTCG
TCACTGCCGACCACTCCCACGTCTTCTCCTTCGGAGGCTACCCCCTGCGAGGGAGCT
CCATCTTCGGGCTGGCCCCTGGCAAGGCCCGGGACAGGAAGGCCTACACGGTCCTC
CTATACGGAAACGGTCCAGGCTATGTGCTCAAGGACGGCGCCCGGCCGGATGTTAC
CGAGAGCGAGAGCGGGAGCCCCGAGTATCGGCAGCAGTCAGCAGTGCCCCTGGACG
AAGAGACCCACGCAGGCGAGGACGTGGCGGTGTTCGCGCGCGGCCCGCAGGCGCAC
CTGGTTCACGGCGTGCAGGAGCAGACCTTCATAGCGCACGTCATGGCCTTCGCCGCC
TGCCTGGAGCCCTACACCGCCTGCGACCTGGCGCCCCCCGCCGGCACCACCGACGC
CGCGCACCCGGGTTACTCTAGAGTCGGGGCGGCCGGCCGCTTCGAGCAGACAGGAG
CAACCAACTTTTCCCTGCTGAAGCAGGCAGGCGACGTGGAGGAGAATCCTGGACCC
ATGGCCCTGTGGATGCGCTTCCTGCCCCTGCTGGCCCTGCTCGTCCTCTGGGAGCCC
AAGCCTGCCCAGGCTTTTGTCAAACAGCACCTTTGTGGTCCTCACCTGGTGGAGGCT
CTGTACCTGGTGTGTGGGGAACGTGGTTTCTTCTACACACCCAAGTCCCGTCGTAAA
AGGGAGGACCCGCAAGTGCCACAACTGGAGCTGGGTGGAGGCCCGGAGGCCGGGG
ATCTTCAGACCTTGGCACTGGAGGTTGCCCGGCAGAAGCGTGGCATTGTGGATCAGT
GCTGCACCAGCATCTGCTCCCTCTACCAACTGGAGAACTACTGCAACTAAACGCGTC
AGACATGATAAGATACATTGATGAGTTTGGACAAACCACAACTAGAATGCAGTGAAA
AAAATGCTTTATTTGTGAAATTTGTGATGCTATTGCTTTATTTGTAACCATTATAAGC
TGCAATAAACAAGTTAACAACAACAATTGCATTCATTTTATGTTTCAGGTTCAGGGGG
AGGTGTGGGAGGTTTTTTAAAGCAAGTAAAACCTCTACAAATGTGGTATGGCTGATT
ATGATCCTGCCTCGCGCGTTTCGGTGATGACGGTGAAAACCTCTGACACATGCAGCT
CCCGGAGACGGTCACAGCTTGTCTGTCC
GGGTAGGGGAGGCGCTTTTCCCAAGGCAGTCTGGAGCATGCGCTTTAGCAGCCCCG
CTGGGCACTTGGCGCTACACAAGTGGCCTCTGGCCTCGCACACATTCCACATCCACC
GGTAGGCGCCAACCGGCTCCGTTCTTTGGTGGCCCCTTCGCGCCACCTTCTACTCCT
CCCCTAGTCAGGAAGTTCCCCCCCGCCCCGCAGCTCGCGTCGTGCAGGACGTGACA
AATGGAAGTAGCACGTCTCACTAGTCTCGTGCAGATGGACAGCACCGCTGAGCAATG
GAAGCGGGTAGGCCTTTGGGGCAGCGGCCAATAGCAGCTTTGCTCCTTCGCTTTCTG
GGCTCAGAGGCTGGGAAGGGGTGGGTCCGGGGGCGGGCTCAGGGGCGGGCTCAGG
GGCGGGGCGGGCGCCCGAAGGTCCTCCGGAGGCCCGGCATTCTGCACGCTTCAAAA
GCGCACGTCTGCCGCGCTGTTCTCCTCTTCCTCATCTCCGGGCCTTTCGACCTGCAG
CCCAAGCTTACCATGGCCAAGTTGACCAGTGCCGTTCCGGTGCTCACCGCGCGCGAC
GTCGCCGGAGCGGTCGAGTTCTGGACCGACCGGCTCGGGTTCTCCCGGGACTTCGT
GGAGGACGACTTCGCCGGTGTGGTCCGGGACGACGTGACCCTGTTCATCAGCGCGG
TCCAGGACCAGGTGGTGCCGGACAACACCCTGGCCTGGGTGTGGGTGCGCGGCCTG
GACGAGCTGTACGCCGAGTGGTCGGAGGTCGTGTCCACGAACTTCCGGGACGCCTC

**CGGGCCGGCCATGACCGAGATCGGCGAGCAGCCGTGGGGGCGGGAGTTCGCCCTGC
GCGACCCGGCCGGCAACTGCGTGCACTTCGTGGCCGAGGAGCAGGACGGAAGCGGA
GCTACTAACTTCAGCCTGCTGAAGCAGGCTGGAGACGTGGAGGAGAACCCTGGACC
TTCCGGAGTGAGCAAGGGCGAGGAGCTGTTCACCGGGGTGGTGCCCATCCTGGTCG
AGCTGGACGGCGACGTAAACGGCCACAAGTTCAGCGTGTCCGGCGAGGGCGAGGGC
GATGCCACCTACGGCAAGCTGACCCTGAAGTTCATCTGCACCACCGGCAAGCTGCCC
GTGCCCTGGCCCACCCTCGTGACCACCCTGACCTACGGCGTGCAGTGCTTCAGCCGC
TACCCCGACCACATGAAGCAGCACGACTTCTTCAAGTCCGCCATGCCCGAAGGCTAC
GTCCAGGAGCGCACCATCTTCTTCAAGGACGACGGCAACTACAAGACCCGCGCCGA
GGTGAAGTTCGAGGGCGACACCCTGGTGAACCGCATCGAGCTGAAGGGCATCGACT
TCAAGGAGGACGGCAACATCCTGGGGCACAAGCTGGAGTACAACTACAACAGCCAC
AACGTCTATATCATGGCCGACAAGCAGAAGAACGGCATCAAGGTGAACTTCAAGATC
CGCCACAACATCGAGGACGGCAGCGTGCAGCTCGCCGACCACTACCAGCAGAACAC
CCCCATCGGCGACGGCCCCGTGCTGCTGCCCGACAACCACTACCTGAGCACCCAGTC
CGCCCTGAGCAAAGACCCCAACGAGAAGCGCGATCACATGGTCCTGCTGGAGTTCG
TGACCGCCGCCGGGATCACTCTCGGCATGGACGAGCTGTACAAGTAA**

pQL452:ITR-P_{PsiR3}-mTSLP-pA::P_{mPGK}-ZeoR-P2A-EGFP-pA-ITR. The grey highlight indicates three copies of the PsiO sequence.

**GACATTGATTATTGACTAGTTATTAATAGTAATCAATTACGGGGTCATTAGTTCATAG
CCCATATATGGAGTTCCGCGTTACATAACTTACGGTAAATGGCCCGCCTGGCTGACC
GCCCAACGACCCCCGCCCATTGACGTCAATAATGACGTATGTTCCCATAGTAACGCC
AATAGGGACTTTCCATTGACGTCAATGGGTGGAGTATTTACGGTAAACTGCCCACTT
GGCAGTACATCAAGTGTATCATATGCCAAGTACGCCCCCTATTGACGTCAATGACGG
TAAATGGCCCGCCTGGCATTATGCCCAGTACATGACCTTATGGGACTTTCCTACTTG
GCAGTACATCTACGTATTAGTCATCGCTATTACCATGGTGATGCGGTTTTGGCAGTA
CATCAATGGGCGTGGATAGCGGTTTGACTCACGGGGATTTCCAAGTCTCCACCCCAT
TGACGTCAATGGGAGTTTGTTTTGGCACCAAAATCAACGGGACTTTCCAAAATGTCG
TAACAACTCCGCCCCATTGACGCAAATGGGCGGTAGGCGTGTACGGTGGGAGGTCT
ATATAAGCAGAGCTCTCTGGCTAACTAGAGAACCCACTGCTTACTGGCTTATCGAAA
TTAATACGACTCACTATAGGGAGACCCAAGCTGGCTAGCGTTTAAACTTAAGCTTGG
TACCATTGCACAATCGATGGTGCAAATTGCACAATCGATGGTGCAAATTGCACAATC
GATGGTGCAAGGCCACCATGGTTCTTCTCAGGAGCCTCTTCATCCTGCAAGTACTAG
TACGGATGGGGCTAACTTACAACTTTTCTAACTGCAACTTCACGTCAATTACGAAAAT
ATATTGTAACATAATTTTTCATGACCTGACTGGAGATTTGAAAGGGGCTAAGTTCGA
GCAAATCGAGGACTGTGAGAGCAAGCCAGCTTGTCTCCTGAAAATCGAGTACTATAC
TCTCAATCCTATCCCTGGCTGCCCTTCACTCCCCGACAAAACATTTGCCCGGAGAAC
AAGAGAAGCCCTCAATGACCACTGCCCAGGCTACCCTGAAACTGAGAGAAATGACG
GTACTCAGGAAATGGCACAAGAAGTCCAAAACATCTGCCTGAATCAAACCTCACAAA
TTCTAAGATTGTGGTATTCCTTCATGCAATCTCCAGAATAAACGCGTCAGACATGATA
AGATACATTGATGAGTTTGGACAAACCACAACTAGAATGCAGTGAAAAAAATGCTTT
ATTTGTGAAATTTGTGATGCTATTGCTTTATTTGTAACCATTATAAGCTGCAATAAAC
AAGTTAACAACAACAATTGCATTCATTTTATGTTTCAGGTTCAGGGGGAGGTGTGGG
AGGTTTTTTAAAGCAAGTAAAACCTCTACAAATGTGGTATGGCTGATTATGATCCTGC
CTCGCGCGTTTCGGTGATGACGGTGAAAACCTCTGACACATGCAGCTCCCGGAGACG
GTCACAGCTTGTCTGTCCGGGTAGGGGAGGCGCTTTTCCCAAGGCAGTCTGGAGCAT
GCGCTTTAGCAGCCCCGCTGGGCACTTGGCGCTACACAAGTGGCCTCTGGCCTCGCA
CACATTCCACATCCACCGGTAGGCGCCAACCGGCTCCGTTCTTTGGTGGCCCCTTCG
CGCCACCTTCTACTCCTCCCCTAGTCAGGAAGTTCCCCCCCGCCCCGCAGCTCGCGT
CGTGCAGGACGTGACAAATGGAAGTAGCACGTCTCACTAGTCTCGTGCAGATGGACA**

GCACCGCTGAGCAATGGAAGCGGGTAGGCCTTTGGGGCAGCGGCCAATAGCAGCTT
TGCTCCTTCGCTTTCTGGGCTCAGAGGCTGGGAAGGGGTGGGTCCGGGGGCGGGCT
CAGGGGCGGGCTCAGGGGCGGGGCGGGCGCCCGAAGGTCCTCCGGAGGCCCGGCA
TTCTGCACGCTTCAAAAGCGCACGTCTGCCGCGCTGTTCTCCTCTTCCTCATCTCCG
GGCCTTTCGACCTGCAGCCCAAGCTTACCATGGCCAAGTTGACCAGTGCCGTTCCGG
TGCTCACCGCGCGCGACGTCGCCGGAGCGGTCGAGTTCTGGACCGACCGGCTCGGG
TTCTCCCGGGACTTCGTGGAGGACGACTTCGCCGGTGTGGTCCGGGACGACGTGAC
CCTGTTCATCAGCGCGGTCCAGGACCAGGTGGTGCCGGACAACACCCTGGCCTGGG
TGTGGGTGCGCGGCCTGGACGAGCTGTACGCCGAGTGGTCGGAGGTCGTGTCCACG
AACTTCCGGGACGCCTCCGGGCCGGCCATGACCGAGATCGGCGAGCAGCCGTGGGG
GCGGGAGTTCGCCCTGCGCGACCCGGCCGGCAACTGCGTGCACTTCGTGGCCGAGG
AGCAGGACGGAAGCGGAGCTACTAACTTCAGCCTGCTGAAGCAGGCTGGAGACGTG
GAGGAGAACCCTGGACCTTCCGGAGTGAGCAAGGGCGAGGAGCTGTTCACCGGGGT
GGTGCCCATCCTGGTCGAGCTGGACGGCGACGTAAACGGCCACAAGTTCAGCGTGT
CCGGCGAGGGCGAGGGCGATGCCACCTACGGCAAGCTGACCCTGAAGTTCATCTGC
ACCACCGGCAAGCTGCCCGTGCCCTGGCCCACCCTCGTGACCACCCTGACCTACGGC
GTGCAGTGCTTCAGCCGCTACCCCGACCACATGAAGCAGCACGACTTCTTCAAGTCC
GCCATGCCCGAAGGCTACGTCCAGGAGCGCACCATCTTCTTCAAGGACGACGGCAA
CTACAAGACCCGCGCCGAGGTGAAGTTCGAGGGCGACACCCTGGTGAACCGCATCG
AGCTGAAGGGCATCGACTTCAAGGAGGACGGCAACATCCTGGGGCACAAGCTGGAG
TACAACTACAACAGCCACAACGTCTATATCATGGCCGACAAGCAGAAGAACGGCATC
AAGGTGAACTTCAAGATCCGCCACAACATCGAGGACGGCAGCGTGCAGCTCGCCGA
CCACTACCAGCAGAACACCCCCATCGGCGACGGCCCCGTGCTGCTGCCCGACAACC
ACTACCTGAGCACCCAGTCCGCCCTGAGCAAAGACCCCAACGAGAAGCGCGATCAC
ATGGTCCTGCTGGAGTTCGTGACCGCCGCCGGGATCACTCTCGGCATGGACGAGCT
GTACAAGTAA
